# Genome-wide DNA methylation profiling in chronic lymphocytic leukaemia

**DOI:** 10.3389/fgene.2022.1056043

**Published:** 2023-01-11

**Authors:** Qiuyi Zhang, Ying Gao, Shuchun Lin, Lynn R. Goldin, Yonghong Wang, Holly Stevenson, Daniel C. Edelman, Keith Killian, Gerald Marti, Paul S. Meltzer, Song Xiang, Neil E. Caporaso

**Affiliations:** ^1^ CAS Key Laboratory of Nutrition, Metabolism and Food Safety, Shanghai Institute of Nutrition and Health, University of Chinese Academy of Sciences, Chinese Academy of Sciences, Shanghai, China; ^2^ Division of Cancer Epidemiology and Genetics, National Cancer Institute, National Institutes of Health, Bethesda, MD, United States; ^3^ Center for Cancer Research, National Cancer Institute, National Institutes of Health, Bethesda, MD, United States; ^4^ Lymphoid Malignancies Section, Hematology Branch, NHLBI, National Institutes of Health, Bethesda, MD, United States; ^5^ Department of Biochemistry and Molecular Biology, School of Basic Medical Sciences, Tianjin Medical University, Tianjin, China

**Keywords:** DNA methyaltion, chronic lymphocytic leukaemia (CLL), illumina 450 K beadchip methylation array, CD19+ B cells, differentially methylated and expressed genes (DMEGs)

## Abstract

**Background:** DNA methylation aberrations are widespread among the malignant B lymphocytes of patients with chronic lymphocytic leukaemia (CLL), suggesting that DNA methylation might contribute to the pathogenesis of CLL.

**Aim:** We aimed to explore the differentially methylated positions (DMPs) associated with CLL and screen the differentially methylated and expressed genes (DMEGs) by combining public databases. We aimed to observe the direction of each DMEG in CLL based on the DMPs in the promoter and the body region respectively to narrow down DMEGs. We also aimed to explore the methylation heterogeneity of CLL subgroups and the effect of B cells maturation on CLL.

**Methods:** In this population-based case control study, we reported a genome-wide DNA methylation association study using the Infinium HumanMethylation450 BeadChip, profiling the DNA methylation of CD19^+^ B Cells from 48 CLL cases and 28 healthy controls. By integrating methylation data and expression data from public databases, gene sets were jointly screened, and then the relationship between methylation sites in promoter and body region and expression of each gene was explored. In addition, support vector machine (SVM) classification algorithm was used to identify subgroups of CLL cases based on methylation pattern, and the effect of B-cell differentiation related methylation sites on CLL-related sites was observed.

**Results:** We identified 34,797 DMPs related to CLL across the genome, most of which were hypomethylated; the majority were located in gene body regions. By combining these DMPs with published DNA methylation and RNA sequencing data, we detected 26,244 replicated DMPs associated with 1,130 genes whose expression were significantly different in CLL cases. Among these DMEGs, nine low expressed DMEGs were selected with hypermethylated in promoter and hypomethylated in body region, and 83 high expressed DMEGs were selected with both hypomethylated in promoter and body region. The 48 CLL cases were divided into 3 subgroups based on methylation site by SVM algorithm. Over 92% of CpGs associated with B cell subtypes were found in CLL-related DMPs.

**Conclusion:** The DNA methylation pattern was altered across the genome in CLL patients. The methylation of *ZAP70*, *FMOD*, and *ADAMTS17* was significantly different between CLL cases and controls. Further studies are warranted to confirm our findings and identify the underlying mechanisms through which these methylation markers are associated with CLL.

## Introduction

Chronic lymphocytic leukaemia (CLL) is characterized by the accumulation of B lymphocytes in the peripheral blood, bone marrow, and secondary lymphoid tissues, and it is the most common adult leukaemia in Western countries (Mansouri et al., 2018). It was estimated that 20,720 Americans would be diagnosed with CLL and 3,930 patients would die of CLL in 2019 (Siegel et al., 2019). At present, there is no efficient early treatment available for this haematologic malignancy, and in most cases, treatments start only when clinical symptoms develop. A clear understanding of the disease pathogenesis could facilitate the prevention and better treatment of CLL.

CLL is a clinically heterogeneous disease, and CLL subtypes differ in terms of B Cell activation, maturation, and cellular subgroup (Chiorazzi et al., 2005). Biological and genetic characteristics such as *ZAP70* expression, immunoglobulin heavy chain variable region (*IGHV*) mutation, and cytogenetic abnormality (del17p) can be linked to CLL prognosis (Parikh and Shanafelt, 2016). A meta-analysis of genome-wide association studies has defined genetic components of CLL aetiology (Berndt et al., 2016). However, the genetic variations identified for CLL in high-risk families can only partially explain CLL risk.

DNA methylation perturbations are common in cancer genomes and even in precancerous normal tissue, playing a pivotal role in the early tumorigenic process (Feinberg, 2018). DNA methylation serves as an intermediate between gene and environmental interactions, study of DNA methylation might explain epigenetic mechanism of CLL pathogenesis. The methylation pattern in CLL patients of peripheral blood and lymph node samples is relatively stable over time, suggesting that aberrant methylation might be involved in the early phase of leukaemogenesis (Cahill et al., 2013). Studies have suggested that DNA methylation changes not only distinguished CLL and healthy control but also exhibited large interpatient differences in CLL, which could be used to distinguish CLL subtypes with different IGHV statuses (Supplementary Table S1). It has been reported that the transcriptional start sites of many tumour suppressor genes (TSGs) are located in CpG islands (CGIs), which are usually hypermethylated in tumours (Herman and Baylin, 2003). Besides the gene silence by hypermethylation of promoter region, altered methylation observed in the gene bodies could also influence gene expression (Kulis et al., 2012; Yang et al., 2014). However, the relationship between DNA methylation and gene expression level in CLL was not consistent, more epigenome-wide association studies, integrating expression data, are needed to explore the role of DNA methylation in the CLL pathogenesis. In addition, CLL heterogeneity and the cell types caused by B Cell differentiation are considered to bias the pathogenesis of CLL. In this study, we also explored the influence of CLL subtypes and the methylation changes related to B Cell differentiation on the methylation change in the case-control screening.

## Materials and methods

### Study population

Participants were recruited from the NCI B-CLL Registry, which has been described previously (Ishibe et al., 2001). Demographic information was collected from surveys and questionnaires. Clinical information was obtained from private physicians, patient interviews, hospital records, death certificates, and NIH clinic visits.

### Sample preparation

Fifty CLL patients, two MBL patients, and 29 unrelated healthy controls were included in the current study. The peripheral blood samples collected closest to diagnosis were used for the assay. B lymphocytes were isolated from cryopreserved peripheral blood lymphocytes by magnetic cell separation (ZenBio Advanced Cell-based Solutions and Services, North Carolina) using a CD19^+^ antibody (Miltenyi Biotec, Bergisch Gladbach, Germany). The cell purity of the isolated samples was evaluated by flow cytometry using propidium iodide for live/dead cell discrimination with CD45/CD19 antigens. Sample aliquots of CD19^+^ B Cells with purity greater than 90% were selected for DNA extraction.

### Microarray-based DNA methylation assay

DNA from the isolated CD19^+^ B Cells was extracted with a DNeasy Blood & Tissue Kit (Qiagen, Valencia, CA). For each sample, 500 ng of genomic DNA was bisulfite converted using the EZ DNA Methylation Kit (Zymo Research, Irvine, CA), and 125 ng bisulfite-converted DNA was used to evaluate methylation levels with Infinium HumanMethylation450K BeadChip (Illumina, Inc, San Diego, CA) according to the manufacturer’s protocol.

### 450k DNA methylation data analysis

Raw 450K BeadChip methylation data were extracted from *GenomeStudio* (Illumina, Inc.). Two samples were excluded due to the high average detection *p*-values (over 1% CpG sites with detection *p* > 0.05). One sample was excluded due to the misclassification of sex by multidimensional scaling (MDS) of CpG sites on the X chromosome. CpG sites were excluded if 1) over 1% of samples had a detection *p* > 0.05; 2) over 5% of samples had bead counts <3; 3) they were located on X or Y chromosomes; or 4) they were annotated with SNPs and cross-reactive probes (Zhou et al., 2017). Data normalization was performed using dasen and beta-mixture quantile normalization. Batch effects were corrected by ComBat (Johnson et al., 2007). Altogether, 406,879 autosomal probes from 76 samples (48 CLL cases and 28 controls) were included in the final analysis. Two MBL samples were used for preliminary exploration.

Linear regression adjusted for age and sex comparing CLL cases and controls was performed using the R package *limma*. Beta values were transformed to M-values for the linear regression model. *p* values were corrected for multiple testing by Benjamini–Hochberg FDR (q-value). Probes with methylation differences over 0.2 (|Δβ|> 0.2) between cases and controls and FDRs less than 0.05 (q-value <0.05) were identified as differentially methylated positions (DMPs). The chi-square goodness of fit test was used to compare the distribution of DMPs with genomic background CpGs. Differentially methylated regions (DMRs) containing more than seven DMPs with lengths over 50 bp were defined by *ProbeLasso* (Butcher and Beck, 2015). The minimum distance between two adjacent DMRs was 1,000 bp.

Copy number aberrations (CNAs) were profiled using the total signal intensity of probes by the Infinium array (Feber et al., 2014). The copy gain or loss on each chromosome was defined using a threshold of 0.2.

### Integration with public DNA methylation and transcription data

The publicly available datasets of DNA methylation and RNA transcription used in this study are shown (Supplementary Table S2A). DNA methylation data of Illumina 450K methylation array from the European Genome-Phenome Archive (EGA), EGAD00010000254 and EGAD00010000871 were requested. The CD19^+^ B Cell samples of 329 CLL cases (139 from EGAD00010000254, 190 from EGAD00010000871) and 21 healthy controls (14 from EGAD00010000254, seven from EGAD00010000871) were finally selected from the dataset of EGAD00010000871 after quality control (Supplementary Table S2B), and their Methylation data were extracted and analysed with the procedure described above for replication. DMPs identified with methylation changes in the same direction as our data were defined as replicated DMPs. RNA sequencing data, including 145 CLL samples (98 from EGAD00001000258, 47 from GSE66117) and seven control samples (2 from GSE62246, five from GSE70830) from the Gene Expression Omnibus, were requested (Supplementary Table S2C). RNA sequencing data were evaluated and merged by *FastQC* and *multiQC*. *Trimmomatic* (Bolger et al., 2014) was used for read filtering. RNA reads were aligned to the human reference genome hg19 (UCSC) using *STAR* (Dobin et al., 2013) and were assembled for quantification using *featureCounts* to generate counts (Liao et al., 2014). Altogether, RNA expression data of CD19^+^ B Cells from 144 CLL and seven controls were included for downstream analysis after filtering one case due to the total counts of genes less than 10 0000. Genes with expression differences larger than 2-fold (|log2fold change| > 1) and Benjamini–Hochberg adjusted *p* values less than 0.05 (q-value <0.05) were defined as differentially expressed genes (DEGs) using DESeq2 (Love et al., 2014). DEGs covered by replicated DMPs were defined as differentially methylated and expressed genes (DMEGs) by combining methylation data in our study and DEG lists analysed with public datasets. Gene Ontology (GO) enrichment analysis and KEGG pathway analysis were performed on DMEGs using *clusterProfiler* (Yu et al., 2012) for enriched biological process (BP), molecular function (MF), and cell component (CC) terms and KEGG pathways. The significantly enriched GO terms and KEGG pathways were identified as those with q-values less than 0.05.

Since DMPs in promoter and gene body region could be associated with gene expression (Kulis et al., 2012), the relationship of DMPs in different regions (promoter and body region) and expression was inconsistent. We conducted integrative analysis by combining DNA methylation data in our study and expression data from public datasets. We defined the promoter region as the combination of TSS1500, TSS200, and the first exon (Jiao et al., 2014). To narrow down the DMEG gene list, we selected the DMPs in the promoter region had a negative direction with expression, and the DMPs in the gene body region had a positive or negative direction with expression; and the overlap genes of these two types of DMPs might be the potential methylation regulated DEGs.

**FIGURE 1:**
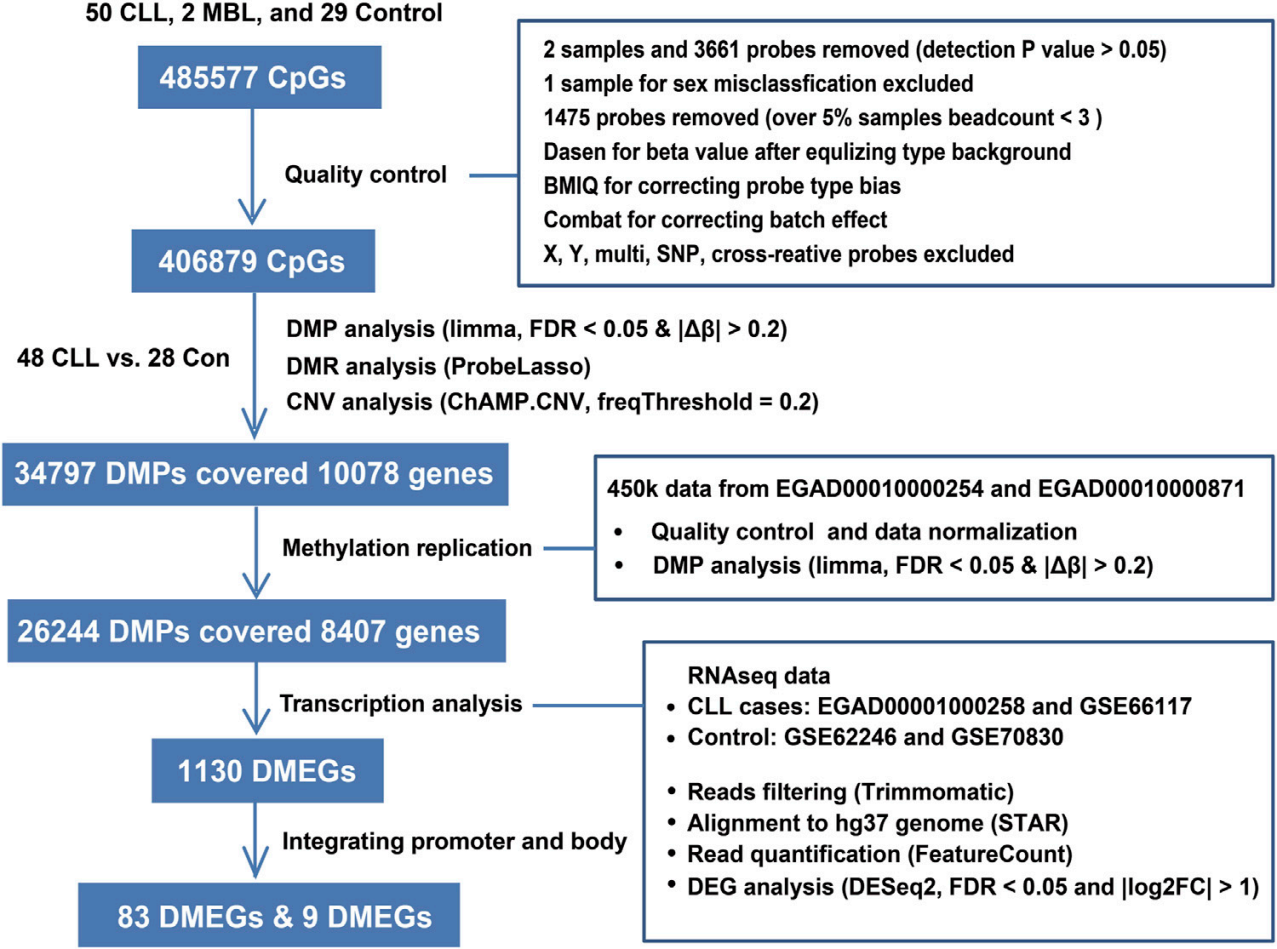
Flowchart showing our steps for identifying methylation markers.

A flow chart describing these analysis steps is shown in Figure 1.

### Methylation of CLL subtypes

Based on IGHV status, CLL can be classified as M-CLL (IGHV mutated) or U-CLL (IGHV unmutated). CLL can also be classified as naive B-cell-like (n-CLL), intermediate (i-CLL), and memory B-cell-like CLL (m-CLL) based on methylation level, where n-CLL and m-CLL shared high similarity with U-CLL and M-CLL, respectively. We applied the support vector machine (SVM) model (C = 10, γ = 0.01) reported to classify the 48 CLL patients in our study into subgroups of m-CLL, n-CLL, and i-CLL based on the five CpGs (cg00869668, cg03462096, cg09637172, cg11472422, cg17014214) selected with 133 CLL cases in the ICGC database reported by Queiros et al. (2015). Linear regression adjusted for sex and age was fitted to find the DMPs between n-CLL and m-CLL. Subtype-related DMPs were compared to CLL-related DMPs to evaluate the impact of CLL methylation heterogeneity on DMP identification.

### Methylation of normal B Cell subtypes

To explore the impact of normal B Cell differentiation on CLL-related DMPs detection, DNA methylation data of 14 samples of CD19^+^ B Cell mixture and three samples of each purified B Cell subtypes, including naive B Cells (NBCs), CD5^+^ naive B Cells (CD5^+^ NBCs), class-switched memory B Cells (csMBCs), and non-class-switched memory B Cells (ncsMBCs) were requested from EGAD00010000254 dataset (Kulis et al., 2012). A linear regression was fitted to identify DMPs between CD19^+^ B Cells (N = 14) and these four subtypes (N = 3 each). DMPs identified in these four comparisons were combined as normal B Cell differentiation related DMPs. After removing these DMPs from the comparison of normal B Cells to the total CpG sites after quality control, a linear model was fitted on CLL patients and healthy controls to find CLL-related DMPs and DMEGs, which should be independent from B Cell maturation. Gene enrichment analysis was performed on these genes to identify relevant biological processes other than B Cell differentiation.

## Results

### Characteristics of 48 CLL patients and 28 healthy controls

Genome-wide DNA methylation status was evaluated in 48 CLL cases and 28 healthy controls (Table 1). CLL patients were on average approximately 4 years older than controls (*p* = 0.015). Both white blood cell counts (WBCs) and absolute lymphocyte counts (ALCs) in CLL cases were significantly higher than those of controls (*p* = 1.2e-7; P = 3e-5). Among CLL cases, 21 were at Rai stage 0, 22 were at Rai stage I/II, and three were at Rai stage III/IV. Two patients with monoclonal B Cell lymphocytosis (MBL), one male and one female, were also included for preliminary exploration.

**TABLE 1:** Characteristics of the 76 subjects in this study.^a^

Characteristics	CLL (N = 48)	Control (N = 28)	*p* ^b^
Sex			
Female	29 (60%)	19 (68%)	0.69
Male	19 (40%)	9 (32%)	
Age ^c^	61 (55–71.5)	57 (42–66)	0.015
WBC (10^9^/L)	29.2 (22.1–53.7)	7.4 (6.1–8.5)	1.2e-7
ALC (10^9^/L)	21.0 (13.9–36.4)	2.0 (1.6–2.4)	3.0e-5
Rai stage^d^			
Low 0)	21 (46%)		
Intermediate (I/II)	22 (48%)		
High (III/IV)	3 (6%)		

^a^ Two MBL, cases were used to explore the tumor progression related issue assuming MBL, as intermediate point between healthy to CLL.

^b^ Continuous variables were expressed as medians (IQRs), and categorical variables are expressed as frequencies (%). *p* values were calculated by using a wilcoxon rank sum test for continuous variables and chi-square test for categorical variables.

^c^ Age was recorded when blood samples were collected. Age of three subjects in the control group was not available.

^d^ Rai stage for two subjects in the CLL, group were not available.

Abbreviations: WBC, white blood cells; ALC, absolute lymphocyte count.

### CLL-related differentially methylated positions (DMPs)

**FIGURE 2:**
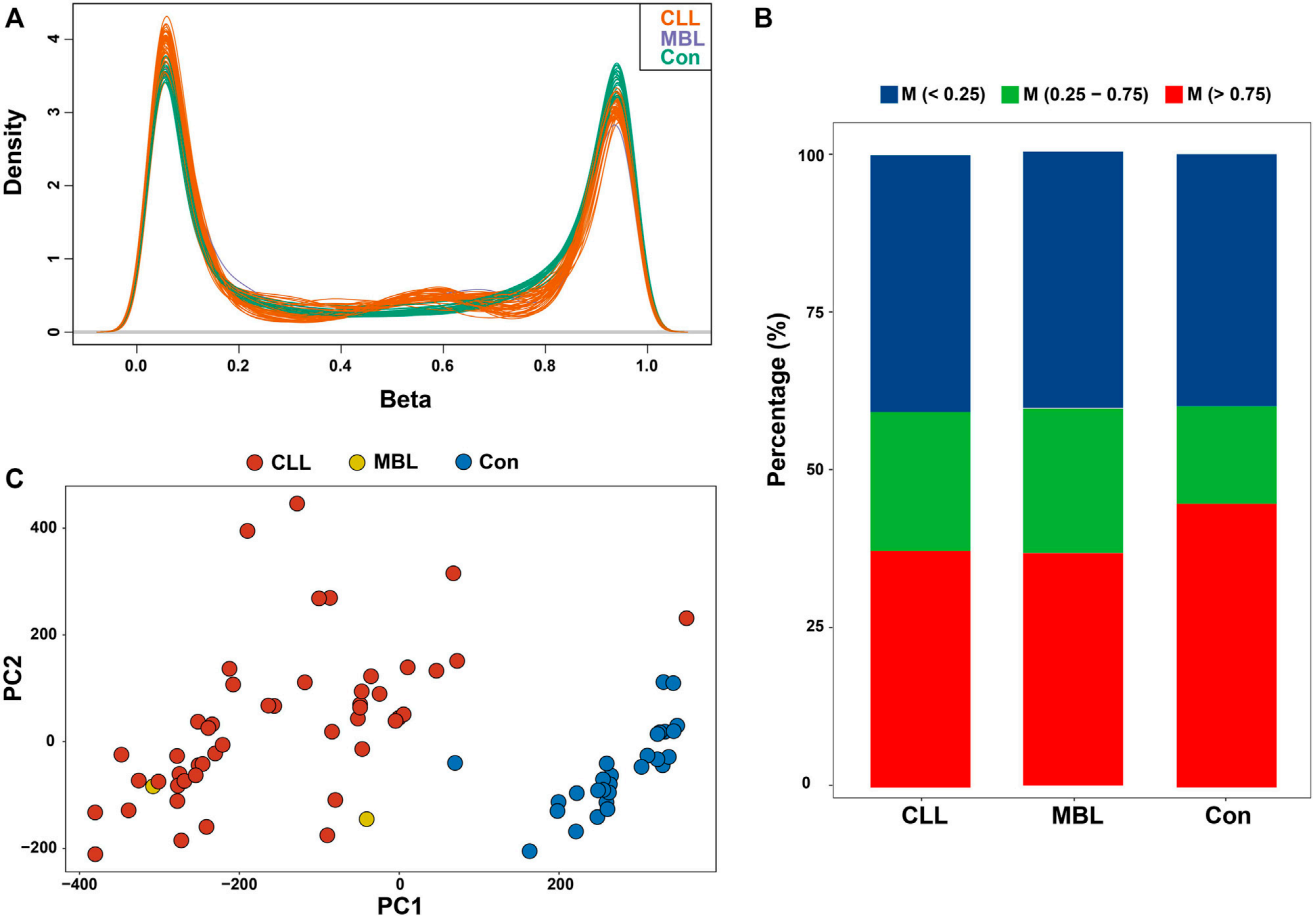
Whole-genome methylation pattern in our study **(A)** Density plot of methylation beta values of 48 CLL patients, two MBL patients, and 28 healthy control after quality control **(B)** Percentage of beta value as three categories (<0.75, 0.25–0.7, and >0.75) in CLL, MBL, and healthy control **(C)** Principal component analysis of all probes (N = 406,879) in CLL, MBL, and control.

**FIGURE 3:**
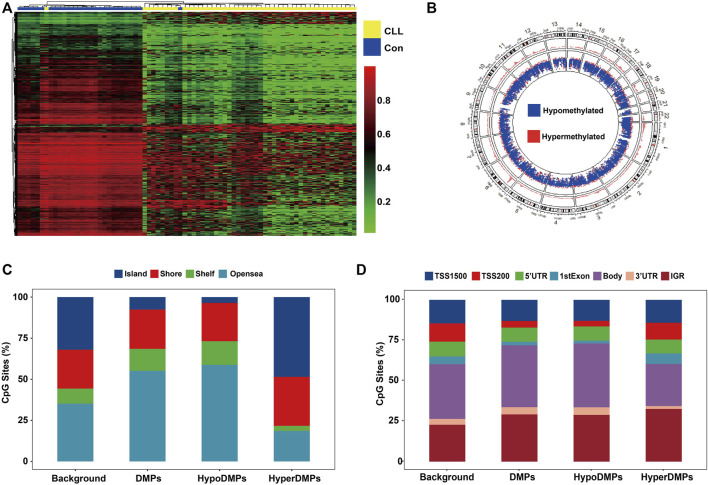
CLL-related DMPs identified in our study **(A)** Hierarchical analysis was performed on the beta value of all CLL-related DMPs (N = 34,797). DMPs with FDR lower than 0.05 were selected adjusted by age and sex using linear regression. Heatmap of CLL-related DMPs showed more perturbed and decreased methylation in CLL patients. **(B)** Density plot of DMP distribution on 22 human autosomes **(C)** Barplots depicting the percentage of DMPs was on each CGI feature (Island, Shore, Shelf, and Opensea). CpG Islands contained more hypermethylated DMPs. Opensea and Shelf regions contained more hypomethylated DMPs. **(D)** Barplots depicting the percentage of DMPs was on each genomic feature (TSS1500, TSS200, 5′UTR, 1stExon, Body, 3′UTR, and IGR). DMPs in the promoter region including the TSS200, TSS1500, 1stExon region showed more hypermethylated. DMPs in the body and 3′ UTR region showed more hypomethylated. Background: Total 406,879 CpGs after quality control; DMPs: Total 34,797 CLL-related DMPs; HyperDMPs: Hypermethylated DMPs in CLL patients; HypoDMPs: Hypomethylated DMPs in CLL patients.

The density plot of beta values showed that genomic CpG sites followed a bimodal distribution in cases and healthy controls (Figure 2A). Compared to controls, both CLL and MBL cases contained more sites with decreased methylation (Figure 2B). Principal component analysis (PCA) based on all probes showed that the overall methylation pattern of CLL cases was significantly different from that of controls, and MBL patients were grouped into CLL cases (Figure 2C). With the criteria of |Δβ| > 0.2 and q-value <0.05, we identified 34,797 DMPs between CLL cases and controls (Supplementary Table S3). Unsupervised clustering analysis showed that most DMPs were hypomethylated in CLL cases compared to controls (Figure 3A). The DMPs were annotated with genomic features and CGI features, and the distribution of DMPs on 22 autosomes is shown in Figure 3B. Over 57.7% of DMPs in CpG islands were hypermethylated (Figure 3C). Nearly 93.9% of DMPs in gene body regions were hypomethylated (Figure 3D). A total of 477 differentially methylated regions (DMRs) located in 232 genes were identified (Supplementary Table S4). Chromosome six contained the highest number of DMRs, with 77 (Supplementary Figure S1).

### CLL-related copy number aberrations (CNAs)

Moreover, 82 CNA regions were detected in CLL cases compared to controls (Supplementary Figure S2; Supplementary Table S5). The copy number loss mapped to chr13, chr17 and chr19 were top three chromosomes. Only three of the 82 CNAs were copy number gain regions, including two regions across *HOXC8* and *GALNT9* on chromosome 12 and one region across *GSTTP1* on chromosome 22. In contrast, 79 copy number loss CNAs were identified; these included two regions across *GSTT1* and *LOC391322* on chromosome 22 that exhibited both copy number gains and losses. The other 77 copy number losses were located on chromosomes 1, 5, 6, 7, 11, 12, 13, 14, 17, and 19.

### Differentially methylated and expressed genes

**FIGURE 4:**
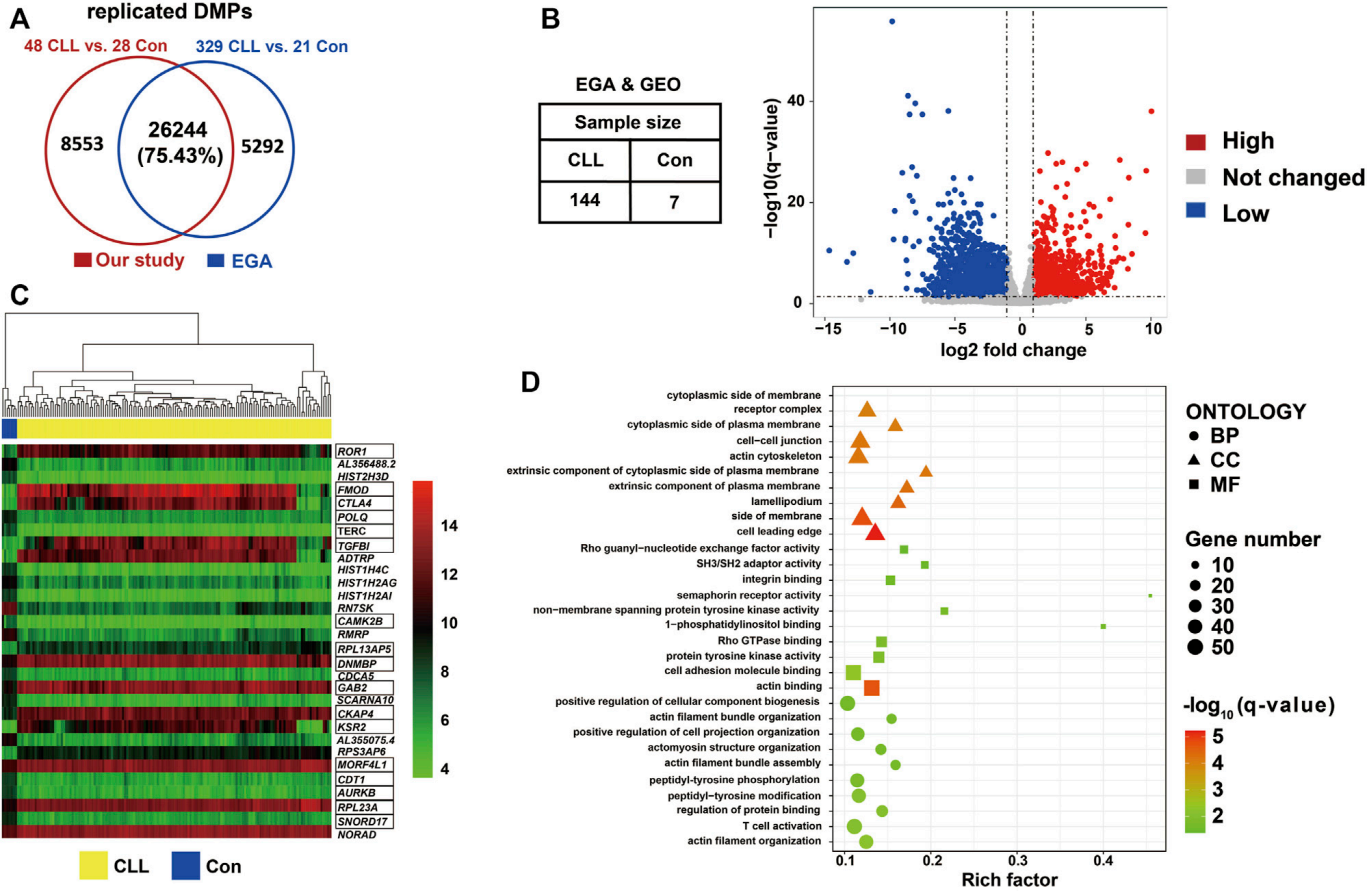
DMEGs identified between CLL patients and controls **(A)** Venn diagram showed the number of replicated DMPs. Replicated DMPs (N = 26,244) overlapped using our dataset of 48 CLL patients and 28 healthy control compared to DMPs identified datasets from EGA of 329 CLL patients and 21 healthy control. More than 75% DMPs from our study were also found in DMPs identified in EGA. **(B)** Volcano plot showed the differentially expressed genes through transcription analysis using DESeq2. Datasets were required from EGA and GEO with a total of 144 CLL patients and seven healthy controls. DEGs (N = 5,104) were selected with the criterion of q-value <0.05 and |foldchange| > 2 using DESeq2. **(C)** Heatmap showed the expression value the top 30 DEGs. The hierarchical clustering analysis was performed using the normalized counts from RNAseq data. The marked genes were in the 1,130 DMEG list. **(D)** Bubble chart shown gene enrichment results of 1,130 DMEGs. Gene ontology terms were shown with the top ten of BP, MF, and CC respectively.

A comparison of 450k methylation data from 329 CLL cases and 21 controls from the EGA revealed 31,536 DMPs. Over 75% of the DMPs found in our study (n = 26,244) could be replicated with the same direction as in the EGA data (Figure 4A; Supplementary Table S6A). We constructed a volcano plot showing the 5,104 differentially expressed genes (DEGs) that were detected by comparing the RNA sequencing data of 144 CLL cases and seven controls from the EGA and GEO datasets (Figure 4B; Supplementary Table S6B); the heatmap of the top 30 DEGs according to normalized counts using vst method was shown in Figure 4C. By combining the DEGs and the replicated DMPs, 1,130 DMEGs were identified (Supplementary Table S6C). We found that *ROR1 and ZAP70* was hypomethylated in the promoter region and highly expressed in CLL patients (Table S6C). The top ten BP, MF, and CC Gene Ontology terms identified in enrichment analysis of these DMEGs (Supplementary Table S7) are shown in Figure 4D. The biological process of actin filament organization and T cell activation were involved in CLL progression.

The DMEGs were classified as three classes: 1.335 DMEGs with DMPs located in gene body regions, and the methylation levels of all these DMPs and gene expression had the same direction in CLL (Supplementary Table S8A). 2.326 DMEGs with DMPs located in body regions, and the methylation levels of all these DMPs and gene expression were in the inverse direction in CLL (Supplementary Table S8B). 3.226 DMEGs with DMPs located in promoter regions, and the methylation levels of all these DMPs and the expression were in the inverse direction (Supplementary Table S8C). Then we combined the DMPs of promoter and body for each DMEG. There were 83 DMEGs whose DMPs were both in body and promoter regions by combining 335 DMEGs and 226 DMEGs. These 83 DMEGs were all highly expressed in CLL with hypomethylated DMPs in the two regions, including *ROR1* and *ZAP70* (Supplementary Table S8D). There were nine DMEGs by combining 326 DMEGs and 226 DMEGs, including *MACROD2*, *ADAMTS17*, *TJP1*, *MET*, *OSBPL1A*, *SYN2*, *KCNG2*, *AGBL4*, and *ME3,* were lower expressed in CLL cases with hypermethylated DMPs in promoter regions and hypomethylated DMP in body regions (Supplementary Table S8E).

### Methylation heterogeneity in CLL

**FIGURE 5:**
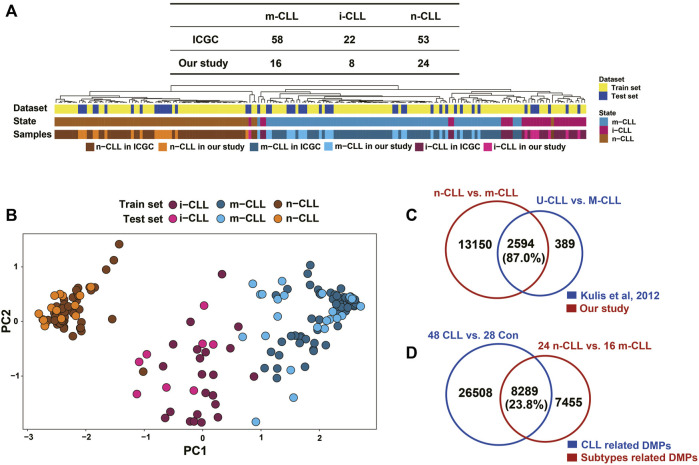
Subgroups in CLL patients classified by methylation pattern. **(A)** Clustering shown SVM modeling of methylation data revealed CLL subgroups in our study. SVM model (Radial Basis Kernel, C = 10, γ = 0.01) was conducted with five CpGs (cg00869668, cg03462096, cg09637172, cg11472422, cg17014214) from were used as train datasets according to *AC Queirós et al, 2014.* CLL patients were classified into three subgroups of 16 m-CLL, eight i-CLL, and 24 n-CLL. **(B)** Principal component analysis on the M value of this five CpGs of 48 CLL patients in our study. Three subgroups of CLL patients could be more closely with the training dataset. **(C)** A comparison of DMPs (N = 15,744) between m-CLL and n-CLL and the previously reported DMPs (N = 3,626) distinguishing M- and U-CLL from Kulis et al., 2012 *.* **(D)** A comparison of the CLL-related DMPs (N = 34,797) identified in our study with DMPs (N = 15,744) distinguishing m-CLL and n-CLL subgroups in our dataset. n-CLL: NBC-like CLLs; m-CLL: MBC-like CLLs.

The 48 CLL patients in our study were classified into subgroups composed of 24 n-CLL, 16 m-CLL, and eight i-CLL patients using the SVM model of five CpG sites reported by Queiros et al. (2015). The hierarchical clustering of the five CpG sites reported in Querios et al., defined as in ICGC and in our study is shown in Figure 5A. PCA also showed that the subgroups were distinct from each other (Figure 5B). By fitting the linear model adjusted for age and sex, 15,744 DMPs covering 5,906 genes were identified by comparing the n-CLL and m-CLL subgroups. It was previously reported that 3,266 DMPs covering 1,519 genes could distinguish the U-CLL and M-CLL subgroups (Kulis et al., 2012); 87% of the 3,266 DMPs covering 1,427 genes also differentiated the n-CLL and m-CLL subtypes in our study (Figure 5C). In addition, compared to healthy controls, only 23.8% of DMPs were overlapped distinguishing the n-CLL and m-CLL subtypes overlapped with the CLL-related DMPs (Figure 5D).

### B Cell differentiation-related methylation changes

**FIGURE 6:**
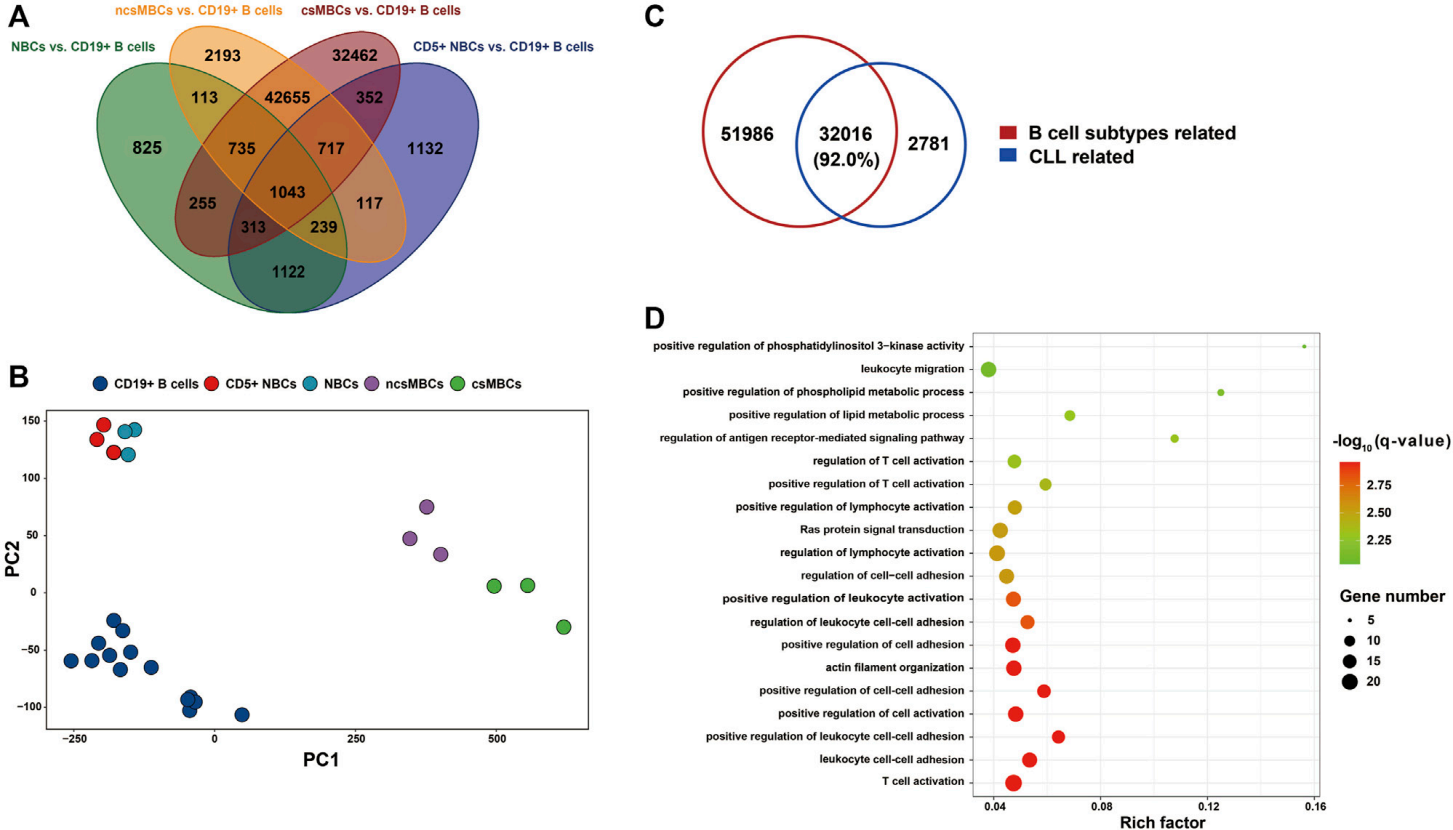
The implication of B Cell differentiation in CLL patients. **(A)** Petal diagram shown DMPs associated with B Cell differentiation (N = 84,273). The DMPs were identified by comparing each B Cell subtype’s DNA methylation with CD19^+^ B Cells mixture, using the dataset from Kulis et al., 2012. **(B)** PCA on the subtypes of normal B Cells based on M value of DMPs associated with B Cell differentiation (N = 84,273). (**C)** CLL-related DMPs (N = 34,797) compared to DMPs associated with B Cell subtypes (N = 84,002). DMPs associated with B Cell subtypes, not in the 406,879 CpGs of the whole genome were removed before integrating. **(D)** Bubble chart shown the top 20 biological processes using enrichment analysis of the DMEGs not associated with B Cell subtypes (N = 274). NBCs: naïve B Cells; CD5^+^ NBCs: CD5^+^ naïve B Cells; csMBC: class-switched memory B Cells; ncsMBCs: non-Class-switched memory B Cells

Studies have shown that dynamic methylation changes during normal B Cell differentiation may play a role in B-CLL aetiology (Kulis et al., 2015; Oakes et al., 2016). To test this hypothesis, we retrieved a 450k DNA methylation dataset for CLL (EGAD00010000254) and compared the DNA methylation patterns of four main CD19^+^ B Cell subtypes (CD5^+^ NBCs, NBCs, ncsMBCs, csMBCs) compared to those of the CD19^+^ B Cell mixture. A total of 84,273 CpGs related to B Cell differentiation subtypes were identified (Figure 6A). The top two principal components of the M-value of these CpGs could also differentiate the subtypes from the CD19^+^ B Cell mixture (Figure 6B). Comparing the B Cell differentiation-related DMPs to the CLL-related DMPs showed that more than 92% of CLL-related DMPs were normal B Cell subtype-related (Figure 6C). After removing the 84,273 CpGs related to B-cell differentiation, 2,781 CpG sites covered by 274 genes out of 34,797 CpG sites with expression changes were detected. The top 20 most enriched biological processes identified by gene enrichment analysis among those 274 genes are shown in Figure 6D. And the enriched terms included T cell activation, cell-cell adhesion, and lipid metabolic process.

## Discussion

In this study, we evaluated 406,879 CpGs and identified 34,797 DMPs related to CLL. More than 90% of the DMPs in CLL patients were hypomethylated and mainly in gene body regions. Combining our study with public datasets of 450k methylation data and RNAseq data, we identified 1,130 DMEGs with methylation and gene expression both altered in CLL patients. Nine low expressed DMEGs were selected with hypermethylated in promoter and hypomethylated in body, such as *ADAMTS17*, and 83 high expressed DMEGs were selected with both hypomethylated in promoter and body, such as *ZAP70*, *FMOD*, *PAX9*. 48 CLL cases were characterized into three subgroups based on SVM model. Methylation sites associated with B Cell subtypes account for large methylation alteration in CLL. Apart from B Cell differentiation, methylation changes in DMEGs involved in T cell activation and lipid metabolism might also contribute to CLL.

Large-scale methylation alterations may contribute to chromosomal instability (Landau et al., 2014; Oakes et al., 2014) and subsequently promote CLL pathogenesis and progression. Losses of 13q and 17p were reported as common cytogenetic aberrations in CLL patients and are associated with the prognosis of CLL (Ouillette et al., 2011; Puiggros et al., 2014). We also observed that the loss of chromosome 13q was the most frequent CNA in CLL patients. The deletion of the 13q14.3 region spans several TSGs, including *FAM10A4* (Sossey-Alaoui et al., 2002), *DLEU7* (Palamarchuk et al., 2010), *KCNRG* (Birerdinc et al., 2010), and *RB1* (Liu et al., 1993), which have been reported to be related to CLL aetiology. A region on chromosome 17q including *MIR21* (17q23.1, 3′ UTR *TMEM49*), one of the microRNA fingerprints in CLL patients (Rossi et al., 2010), was lost in more than 50% of the CLL cases in our study.

There were 34,797 DMPs related to CLL, of which more than 90% were hypomethylated in CLL patients, and mainly located in gene body regions. The gene body regions contained a large number of hypomethylated DMPs in CLL, consistent with a recent study exploring the global distribution of DNA hydroxymethylation and methylation in CLL (Wernig-Zorc et al., 2019).

Combining our study with public datasets of 450k methylation data and RNAseq data, we identified 1,130 DMEGs associated with CLL. Among them, *PAX9* (Rani et al., 2017), *FMOD* (Beekman et al., 2018), and *LEF1* (Beekman et al., 2018) were reported previously. *FMOD* was the top DMEGs with expression level increased more than a thousand folds in CLL patients, which was consistent with report of Beekman et al. (Beekman et al., 2018). *FMOD* belongs to the family of small interstitial proteoglycans, and its silencing could induce apoptosis in CLL cell lines (Choudhury et al., 2010). *SNORD17* the DMEG with the lowest q-value in our study, was reported to play a role in the biogenesis of small nuclear RNAs (snRNAs). *SNORD17* can be regulated by the non-coding splicing factor *SF3B1*, which is frequently mutated in CLL (Shuai et al., 2019). The methylation status of *ZAP70* has been reported to be associated with its expression and has been recognized as a strong prognostic marker for CLL (Corcoran et al., 2005). We found that *ZAP70* was both hypomethylated in the promoter and body region, and was highly expressed in CLL patients. Similarly, the CD5 antigen has been reported to be highly expressed in CLL patients (Bashford-Rogers et al., 2017), and we also found that it was hypomethylated in promoter and body regions and highly expressed in CLL cases. The tyrosine kinase receptor *ROR1* gene, which is involved in the peptidyl-tyrosine modification pathway, was reported to be upregulated in CLL patients (Hojjat-Farsangi et al., 2015). Consistent with this report, we observed hypomethylation of *ROR1* both in promoter and body region and higher *ROR1* expression with 32-fold in CLL patients. *CTLA-4*, a gene that serves as an immune checkpoint to regulate T cell function (Palma et al., 2017), was hypomethylated in the first exon region and body region in our CLL cases and had 128-fold higher expression in CLL patients than in healthy controls according to publicly available transcription data.

Human MHC class II molecules (HLA-DMB, HLA-DMA, HLA-DRA, HLA-DOA) in the top 30 DMRs, which could attract inflammatory tumour-specific CD4^+^ T cells and dampen CD8^+^ T cell antitumour reactivity (Donia et al., 2015), were found to be hypomethylated in all CLL patients in our study. Considering that T cell activation was among the top two enriched biological processes in CLL samples, these results suggest that T cell-related pathways may play an important role in CLL pathogenesis. The cytoskeleton plays a key role during several stages of B Cell activation (Kuokkanen et al., 2015). We observed three actin filament processes among the top ten enriched biological processes in our gene enrichment analysis. One study in 1986 had reported that cytoskeletal organization was aberrantly rearranged with adhesive properties in CLL (Caligaris-Cappio et al., 1986).

There were nine DMEGs were lower expressed in CLL patients, with DNA hypermethylated in the promoter region and hypomethylated in gene body regions, including *MACROD2*, *ADAMTS17*, *TJP1*, *MET*, *OSBPL1A*, *SYN2*, *KCNG2*, *AGBL4*, and *ME3*. Among these genes, *ADAMTS17* was also reported to be hypermethylated and transcriptionally silenced in CLL (Barrow et al., 2021). Although the other eight genes have not been reported in CLL, aberrant methylation, transcription, or genetic alteration of these genes has been observed in various cancers, including breast cancer (Mohseni et al., 2014), colorectal cancer (Thorsen et al., 2011; Lin et al., 2015; Kordowski et al., 2018), prostate cancer (Devaney et al., 2013), malignant glioma (Milinkovic et al., 2013), and some other epithelial cancers (Martin and Jiang, 2009; Kordowski et al., 2018).

By comparing DMPs between m-CLL and n-CLL (classified by SVM model) with CLL-related DMPs, only 23.8% of the CLL-related DMPs we identified were associated with CLL subtypes. This might suggest that the heterogeneity of CLL had a limited impact on the detection of CLL-related DMPs. Using the DNA methylation of B Cell subtypes reported by Kulis et al. (2012), we found that 92% of CLL-related DMPs were involved in the B Cell differentiation process, which suggests that B Cell maturation may play a prominent role in CLL pathogenesis. There were 274 DMEGs identified by comparing CLL and controls after removing the B Cell maturation-related DMPs from background CpG sites. Gene enrichment analysis of the 274 DMEGs suggested that T cell activation and cell adhesion related processes might be involved in the pathogenesis of CLL. Ras protein signal transduction, which has been recognized as an attractive therapeutic target for myeloid leukaemia (Willman, 2001), was also observed as an enriched pathway. Interestingly, the lipid metabolism pathway was also one of the top 20 enriched biological processes. We also found that the gene expression of lipoprotein lipase (LPL) and CD36 were significantly upregulated in CLL patients. These two proteins could activate the uptake of fatty acids and were highly expressed in a lipogenic cancer model (Zaidi et al., 2013). LPL expression was reported to be closely correlated with IGHV mutational status and may be involved in *BCR* and *NOTCH1*-dependent signalling pathways (Bilous et al., 2019). Further studies are needed to decipher the role of lipid metabolism in CLL aetiology. We thought these findings might shed light on finding the new biological process for CLL carcinogenesis.

A previous study showed that methylation status was stable during CLL progression for 6.8 years (Cahill et al., 2013), suggesting that methylation is altered in a very early stage of neoplastic transformation. MBL is defined as the presence of CLL phenotypic cells in the peripheral blood in the absence of other features of CLL. MBL can be categorized as either low-count or high-count based on whether the B Cell count is above or below 0.5×10^9^/L. High-count MBL progresses to CLL requiring therapy at a rate of 1%–2% per year (Strati and Shanafelt, 2015). Therefore, we examined two high-count MBL cases for preliminary exploration. The methylation patterns of these two MBL cases were more similar to that of CLL cases than that of healthy controls, supporting the hypothesis that methylation alteration occurs in a very early stage of CLL development. Since the sample size of MBL was limited in our study, further complementary longitudinal studies of MBL are warranted to validate our findings.

Our study presents several strengths. First, 94% of CLL cases in our study were in the early stage (0, I, II). Thus, the influences of CLL progression and medical treatment on methylation status were limited in these early stage patients compared to late-stage (III and IV) patients. Second, we integrated our data with publicly available methylation and expression data, which provided independent validation of the methylation results and provided with transcription data. There are also limitations in our study. We could not directly validate the DNA methylation and RNA expression for the top DMEGs with our population due to the lack of biospecimens. However, we acquired only 450k DNA methylation data from public databases to validate our findings. The larger population from the public data may provide extra information for methylation pattern and also made up the sample size limited by our own biospecimen.

## Conclusion

In summary, our study revealed that the DNA methylation pattern was altered across the genome in CLL patients. Differentially expressed genes with changes in methylation status, were detected by combining publicly available DNA methylation and RNA expression data. CLL-related DMEGs reported previously, including *ZAP70*, *FMOD*, and *ADAMTS17,* were also detected in our study. Our study also found that the methylation heterogeneity of CLL, and abnormal methylation of B Cell differentiation should be an important factor in the pathogenesis of CLL. Apart from B Cell differentiation, we detected DMEGs involving T cell activation, cell adhesion, Ras signalling, and lipid metabolism were associated with CLL, suggesting that these pathways might contribute to CLL pathogenesis. Further longitudinal large-scale population studies are warranted to replicate our results. Subsequent studies are also needed to decipher the mechanism of these DMEGs in CLL pathogenesis.

## Data Availability

The 450K methylation datasets presented in this study can be found in online repositories. The names of the repository/repositories and accession number(s) can be found below: https://www.biosino.org/node/, OEP000173.

## Supplementary material

Supplementary Figure S1 Chromosome six contains the highest number of DMRs.

Supplementary Figure S2 Composite CNV analysis of 48 CLL cases compared to 28 reference controls based on 450k microarray data.

Supplementary Table S1 Summary of previously reported genome-wide DNA methylation associated studies on CLL.

Supplementary Table S2
**(A)** Public datasets used for methylation replication and transcription analysis in this study. **(B)** Clinical characteristics of samples used for methylation replication (EGAD00010000871 and EGAD00010000254). **(C)** RNASeq data of CLL cases (EGAD00001000258 and GSE66117), and healthy control (GSE62246 and GSE70830).

Supplementary Table S3 CLL-related DMPs (N = 34,797).

Supplementary Table S4 CLL-related DMRs (N = 477).

Supplementary Table S5 CLL-related CNVs (N = 82).

Supplementary Table S6
**(A)** Replicated DMPs (N = 26,244). **(B)** CLL-related DEGs (N = 5,104). **(C)** CLL-related DMEGs (N = 1,130).

Supplementary Table S7 GO enrichment results of 1,130 DMEGs in BP, MF, and CC.

Supplementary Table S8
**(A)** DMEGs with DMPs in body regions all positive with expression (N = 335). **(B)** DMEGs with DMPs in body regions all negative with expression (N = 326). **(C)** DMEGs with DMPs in promoter regions all negative with expression (N = 225). **(D)** DMEGs with DMPs both in promoter and body regions all negative with expression (N = 83). **(E)** DMEGs with DMPs in promoter negative and body regions positive with expression (N = 9).

## Abbreviations

CLL, chronic lymphocytic leukemia; IGHV, immunoglobulin heavy chain variable region; TSGs, tumor suppressor genes; MBL, monoclonal B-cell lymphocytosis; DMPs, differentially methylated positions; DMRs, differentially methylated regions; CNAs, copy number aberrations; PCA, principal component analysis; DEGs, differentially expressed genes; DMEGs, differentially methylated and expressed genes; SVM, support vector machine
