# Supplementary figures and images for: Genome-wide DNA methylation profiling in chronic lymphocytic leukaemia
Source: Front Genet. 2023 Jan 11;13:1056043. doi: 10.3389/fgene.2022.1056043 (PMC9873975; doi:10.3389/fgene.2022.1056043)

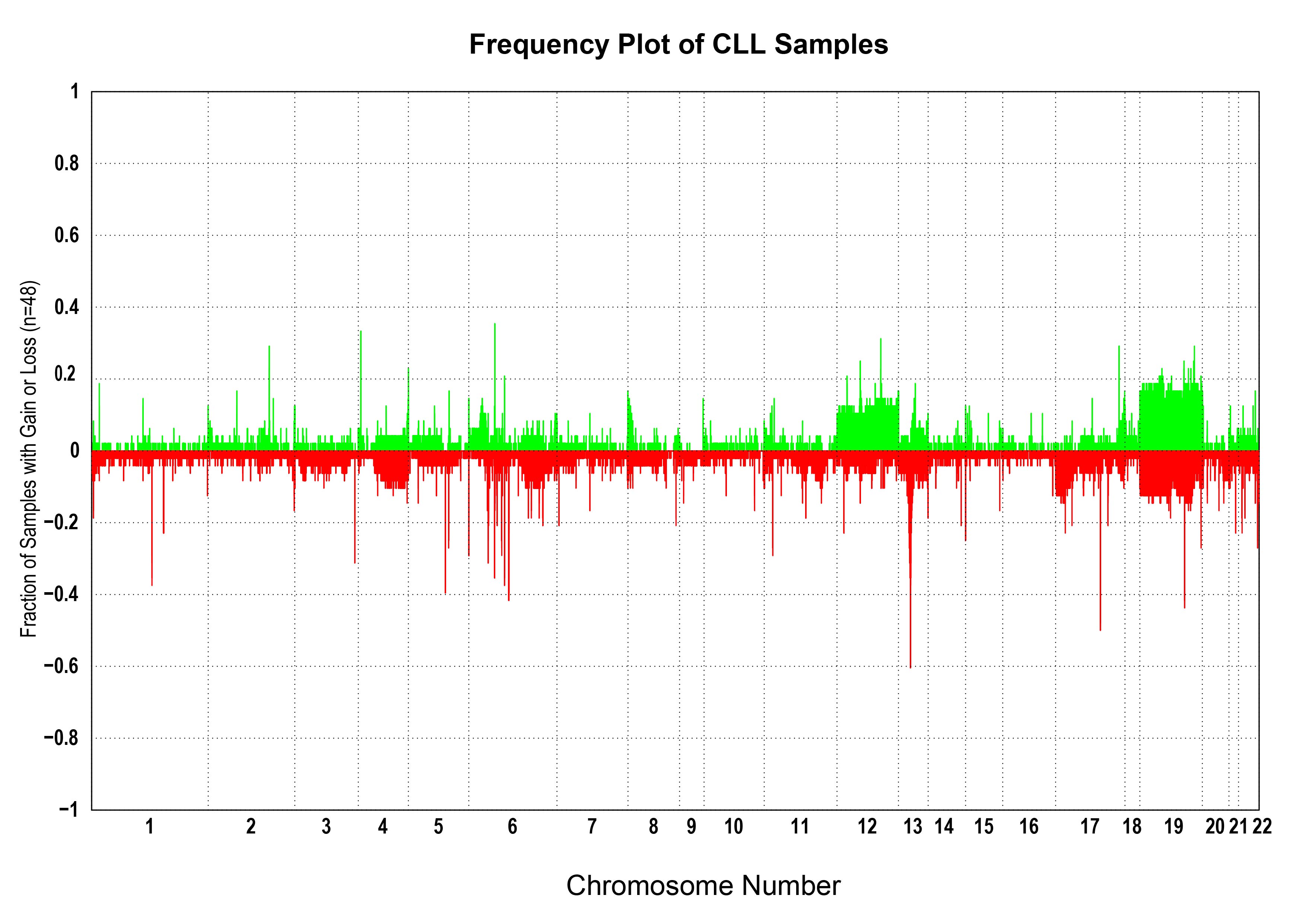

Supplement: Supplementary file 2 [file Image2.jpg]

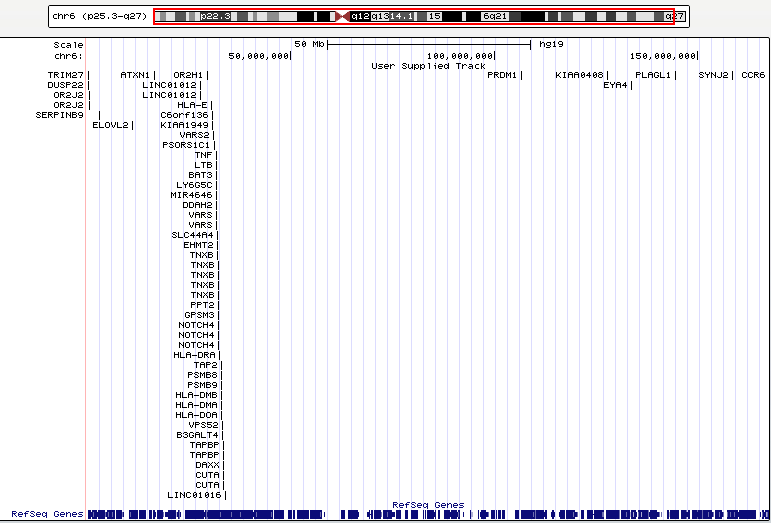

Supplement: Supplementary file 7 [file Image1.png]
