# Supplementary material for: Genome-wide DNA methylation profiling in chronic lymphocytic leukaemia
Source: Front Genet. 2023 Jan 11;13:1056043. doi: 10.3389/fgene.2022.1056043 (PMC9873975; doi:10.3389/fgene.2022.1056043)
Supplement: Supplementary file 5 [file Table1.doc]

Supplementary Table S1. Summary of previously reported genome-wide DNA methylation associated studies on CLL

| Study^a^ | Population source (region)^b^ | Sample size | B cell subtypes | With prior treatment | Stage | EWAS platform^c^ | Genes with methylation changes |
| --- | --- | --- | --- | --- | --- | --- | --- |
| Case control studies on CLL and control | | | | | | | |
| Rush *et al.*, 2004[1]* | CRC (USA) | CLL (10); healthy control (2) | CD19+ | 5 | Not shown | RLGS | *GRM7*, *DERMO1*, *FOXE1*, *TBX3*, *IPF1*, *TBR1*, *GLRB* , *PAK5* |
| Tong *et al.*, 2010[2] | CRC (USA) | CLL (78); healthy control (10) | CD19+ | 11 | Not shown | MCA; promoter microarray | *SOX11*, *DLX1*, *FAM62C*, *SOX14*, *RSPO1*, *ADCY5*, *HAND2*, *SPOCK*, *MLL*, *ING1*, *PRIMA1*, *BCL11B*, *LTBP2*, *BNC1*, *NR2F2*, *SALL1*, *GALGT2*, *LHX1*, *DLX4*, *KLK10*, *TFAP2*, *APP* |
| Pei *et al.*, 2012[3] | Ellis Fischel Cancer Center, (USA)/ the GHSU Cancer, (USA)/ North Shore-LIJ Health | CLL (11); healthy control (3) | CD19+, CD19+/CD27+ MBCs, CD19+/lgD+ NBCs | Not shown | Not shown | RRBS | *FOXD3*, *FOXE1*, *FOXG1*, *IRX1*, *ID4*, *SFRP1*, *SLIT2*, *BNC1*, *ADCY5*, *EBF3*, *NR2F2*, *DIO3*, *FOXA2*, *SOX11*, *BCR*, *LFNG*, *NOTCH1*, *TCF7*, *RASGRF1*, *VAV2*, *NFATc1* |
| Baer *et al.*, 2012[4]* | University Hospital Cologne (Germany) | CLL (24); healthy control (10) | CD19+ | Not shown | Not shown | MCIp | mir-9-2, mir-124-2, mir-129-2, mir-551b, mir-708, mir-124-2, mir-29a/b-1, mir-34a, mir-155, mir-574, mir-1204 |
| Kulis *et al.*, 2012[5]* | ICGC (Spain) | CLL (139); healthy control (14) | CD19+, CD19+CD27+IgM+IgD+ncsMBCs, CD19+CD27+IgA+ /IgG+csMBCs, CD19+CD5+CD27–CD38^low^CD5+ NBCs, CD19+CD27−IgD+NBCs | Not shown | Rai 0 (97), Rai I-II (40), Rai III-IV (2) | Illumina 450K beadchip; WGBS | *PTPRC*, *ASXL1*, *BRAF*, *CDH23*, *EGR2*, *PHC2*, *FAM117A*, *POT1*, *SF3B1* |
| Cahill *et al.*, 2013[6]* | SCALE (Denmark, Sweden) | CLL (18); control(3) | CD19+ | 0 | Not shown | Illumina 450K beadchip | *IBTK*, *LEF1*, *TCF3 ZAP70*, *CLLU1*, *LPL*, *NOTCH1*, *DNMT3B*, *HDAC4*, *HDAC9*, *ABI3*, *WISP3*, *CD86*, *CD80*, *BCL2L1*, *SPN*,*FAS*, *MYB*, *TGFB2*, *TGFBR3*, *ACVR1*, *ZFYVE9*, *SMAD7*, *SMAD3*, *TNF*, *TNFRSF1A*, *TNFRSF8*, *TRAF3IP2*, *NFKB1*, *PARP1*, *RIPK1*, *RIPK3*, *DFFA*, *C1QTNF8* |
| Oakes *et al.*, 2014[7]* | DFKZ (Germany) | CLL (68); Healthy control (11) | CD19/CD20+ | 38 | Not shown | Illumina 450K beadchip | *H3FSA*, *SF3B1*, *IDH1/2*, *TP53* |
| Landau *et al.*, 2014[8]* | USA | CLL (104); Healthy control (26) | CD19+ | 82 | Rai 0-1 (78); Rai 2-4 (26) | WGBS; RRBS | *WIF1*, *DUSP22*, *DCC* |
| Subhash *et al.*, 2016[9] | Sweden | CLL (10)；Healthy control (2) | CD19+ | 0 | Not shown | MBD-Seq | *CRNDE*, *AC012065.7* |
| Rani *et al.*, 2017[10] | India | CLL (14); Healthy control (10) | PBMC (CLL); CD19+ (Control) | 0 | Rai 0-II (14) | Methylated CpG island microarrays | *KLF4*, *PTCH1*, *PAX5*, *PCDH10*, *RASSF10*, *IRX1*, *TBX5*, *ID4*, *SOX7*, *SLIT2*, *TWIST1*, *KLF4*, *TAL1*, *PAX2*, *PAX9*, *NR2F2*, *IRX4*, *MEIS1* |
| Beekman *et al.*, 2018[11] | ICGC( Spain) | CLL (7); Healthy control (15) | CD19+ (CLL);  CD19+lgD+CD27-NBCs,CD19+lgM+/lgD+ or lgA+/lgG+ CD27+MBCs | 0 | Not shown | WGBS | *EBF128*, *FMOD*, *TCF4* |
| Wernig-Zorc *et al.*, 2019[12] | Sahlgrenska University Hospital (Sweden) | CLL (16); healthy control (4) | CD19+;  CD19+CD27-lgM low Naïve B cells, CD19+CD27+IgM+ncsMBCs,CD19+CD27+IgM−csMBCs | 6 | Not shown | MEDIP-seq;  HPLC‑SRM‑MS | Global loss of 5-hmC, significant increase at the gene regulatory regions. *NSMCE1*, *TUBGCP6*, *TUBGCP3* showed higher 5-hmC levels |
| Lin *et al.*, 2019[13] | National Cancer Institute Familial B-cell Registry (USA) | CLL (48); Healthy control (28) | CD19+ | 0 | Rai 0 (21), Rai I-II (22), Rai III-IV (3) | Illumina 450K beadchip | *TRIB1*, *USP9X*, *MED14*, *SYAP1*, *TRAPPC2*, *CA5B*, *EIF1AX*, *STS* |
| Case only studies on CLL cases | | | | | | | |
| Kanduri *et al.*, 2010[14]* | Uppsala University Hospital(Sweden) | Case only (23) | CD19+ | 15 | Binet A (9), Binet B (5), Binet C (3) | Illumina 27K beadchip | IGHV mutated (6) vs unmutated (7):  *ABI3*, *SCGB2A1*, *VHL*, *PPP1R3A*, *WISP3*, *ADORA3*, *AIRE*, *CARD15*, *FABP7*, *LOC340061*, *PRF1*, *UNC5CL*, *ANGPT2*, *IFNB1*, *URP2*, *BCL2*, *PLD1*, *BCL10*, *IL19*, *NGFR* |
| Smith *et al.*, 2015[15]* | CRC (USA) | Case only (27) | CD5+/CD19+ | 26 | Not shown | Illumina 450K beadchip | Recurrent DNA methylation changes during progression at 4752 CpGs enriched for regions near *PRC2* targets as the same direction form serial CLL samples. |
| Oakes *et al.*, 2016[16]* | DFKZ(Germany); ICGC( Spain) | Case only (267) | CD23+IgDhighCD27−NBCs, IgM+IgDlowCD27−CD23−Rhodamine123+MBCs, IgM+IgD+CD27+MBCs, IgG+CD27+MBCs | Not shown | Not shown | WGBS; Illumina 450K beadchip | CLLs derive from normal B-cell development, and CLL subtypes (LP-CLL, IP-CLL and HP-CLL) based on methylation of maturation states link to CLL outcome. TFs (EGR, NFAT, EBF, AP-1) dysregulated in CLL. |
| Papakonstantinou N *et al.*2019 [17] | 9 collaborating institutions in Greece, Germany, Sweden, Italy, France, the Czech Republic and the United Kingdom | Case only (54) | CD19+ B cells | 13 stereotyped subset #8 cases, 13 stereotyped subset，#6 cases and 28 nonsubset U-CLL cases | Not shown | Illumina 450K beadchip | *C6orf150*, *TP63*, *TBC1D22A*, *SDK1*, *GLCCI1*, *ATP5F1*, *WDR77*, *RUNX3*, *BRP44L*, *ETV6*, *TP63* |
| Tsagiopoulou M *et al.*,2019[18] | 9 collaborating institutions in Greece, Germany, Sweden, Italy, France, the Czech Republic and the United Kingdom | Case only (34) | CD19+ B cells | 34 CLL cases who received chemoimmunotherapy as first-line treatment: Of these, 31 were treated with the FCR regimen whereas one each of the remaining 3 cases received FC (fludarabine-cyclophosphamide), FCMR (fludarabine-cyclophosphamide-rituximab + mitoxantrone), or BR (bendamustine-rituximab) | Not shown | Illumina 450K beadchip | Several TFs relevant to B cell/ CLL biology, including members of AP-1, GATA, IRF, POU, NFAT, STAT, and TCF families, as well as most members of *HOX* and *FOX* development-related TFs. |
| Barrow TM et al.,2021*[19]* | Newcastle cohort | Case only (24) | Mononuclear cells | Paired samples from 20 of the patients undergoing treatment and 4 who remained untreated | Not shown | Illumina 450K beadchip | Methylation of the *HOXA4*, *MAFB* and *SLCO3A1* DMRs was associated with post-treatment patient survival. |

a Studies marked by “*” were reviewed by Mansouri et al, 2018.[20]

b CRC, CLL Research Consortium; ICGC, International Cancer Genome Consortium; SCALE, Scandinavian Lymphoma Etiology Study; DFKZ, German Cancer Research Center;

c RLGS, Restriction Landmark Genomic Scanning; MCA, methylated CpG island amplification; RRBS, reduced representation bisulfite sequencing; MCIp, Methyl-CpG immunoprecipitation; WGBS, whole-genome bisulfite sequencing; MBD-seq, Methyl-CpG-binding domain protein enriched genome-wide sequencing; MEDIP-seq, Methylated DNA immunoprecipitation sequencing; HPLC‑SRM‑MS, liquid chromatography tandem‑mass spectrometry

Reference list:

1. Rush LJ, Raval A, Funchain P, Johnson AJ, Smith L, Lucas DM, Bembea M, Liu TH, Heerema NA, Rassenti L *et al*: **Epigenetic profiling in chronic lymphocytic leukemia reveals novel methylation targets**. *Cancer Res* 2004, **64**(7):2424-2433.

2. Tong WG, Wierda WG, Lin E, Kuang SQ, Bekele BN, Estrov Z, Wei Y, Yang H, Keating MJ, Garcia-Manero G: **Genome-wide DNA methylation profiling of chronic lymphocytic leukemia allows identification of epigenetically repressed molecular pathways with clinical impact**. *Epigenetics* 2010, **5**(6):499-508.

3. Pei L, Choi JH, Liu J, Lee EJ, McCarthy B, Wilson JM, Speir E, Awan F, Tae H, Arthur G *et al*: **Genome-wide DNA methylation analysis reveals novel epigenetic changes in chronic lymphocytic leukemia**. *Epigenetics* 2012, **7**(6):567-578.

4. Baer C, Claus R, Frenzel LP, Zucknick M, Park YJ, Gu L, Weichenhan D, Fischer M, Pallasch CP, Herpel E *et al*: **Extensive promoter DNA hypermethylation and hypomethylation is associated with aberrant microRNA expression in chronic lymphocytic leukemia**. *Cancer Res* 2012, **72**(15):3775-3785.

5. Kulis M, Heath S, Bibikova M, Queiros AC, Navarro A, Clot G, Martinez-Trillos A, Castellano G, Brun-Heath I, Pinyol M *et al*: **Epigenomic analysis detects widespread gene-body DNA hypomethylation in chronic lymphocytic leukemia**. *Nat Genet* 2012, **44**(11):1236-1242.

6. Cahill N, Bergh AC, Kanduri M, Goransson-Kultima H, Mansouri L, Isaksson A, Ryan F, Smedby KE, Juliusson G, Sundstrom C *et al*: **450K-array analysis of chronic lymphocytic leukemia cells reveals global DNA methylation to be relatively stable over time and similar in resting and proliferative compartments**. *Leukemia* 2013, **27**(1):150-158.

7. Oakes CC, Claus R, Gu L, Assenov Y, Hullein J, Zucknick M, Bieg M, Brocks D, Bogatyrova O, Schmidt CR *et al*: **Evolution of DNA methylation is linked to genetic aberrations in chronic lymphocytic leukemia**. *Cancer Discov* 2014, **4**(3):348-361.

8. Landau DA, Clement K, Ziller MJ, Boyle P, Fan J, Gu H, Stevenson K, Sougnez C, Wang L, Li S *et al*: **Locally disordered methylation forms the basis of intratumor methylome variation in chronic lymphocytic leukemia**. *Cancer Cell* 2014, **26**(6):813-825.

9. Subhash S, Andersson PO, Kosalai ST, Kanduri C, Kanduri M: **Global DNA methylation profiling reveals new insights into epigenetically deregulated protein coding and long noncoding RNAs in CLL**. *Clinical epigenetics* 2016, **8**:106.

10. Rani L, Mathur N, Gupta R, Gogia A, Kaur G, Dhanjal JK, Sundar D, Kumar L, Sharma A: **Genome-wide DNA methylation profiling integrated with gene expression profiling identifies PAX9 as a novel prognostic marker in chronic lymphocytic leukemia**. *Clinical epigenetics* 2017, **9**:57.

11. Beekman R, Chapaprieta V, Russinol N, Vilarrasa-Blasi R, Verdaguer-Dot N, Martens JHA, Duran-Ferrer M, Kulis M, Serra F, Javierre BM *et al*: **The reference epigenome and regulatory chromatin landscape of chronic lymphocytic leukemia**. *Nat Med* 2018, **24**(6):868-880.

12. Wernig-Zorc S, Yadav MP, Kopparapu PK, Bemark M, Kristjansdottir HL, Andersson PO, Kanduri C, Kanduri M: **Global distribution of DNA hydroxymethylation and DNA methylation in chronic lymphocytic leukemia**. *Epigenetics Chromatin* 2019, **12**(1):4.

13. Lin S, Liu Y, Goldin LR, Lyu C, Kong X, Zhang Y, Caporaso NE, Xiang S, Gao Y: **Sex-related DNA methylation differences in B cell chronic lymphocytic leukemia**. *Biology of sex differences* 2019, **10**(1):2.

14. Kanduri M, Cahill N, Goransson H, Enstrom C, Ryan F, Isaksson A, Rosenquist R: **Differential genome-wide array-based methylation profiles in prognostic subsets of chronic lymphocytic leukemia**. *Blood* 2010, **115**(2):296-305.

15. Smith EN, Ghia EM, DeBoever CM, Rassenti LZ, Jepsen K, Yoon KA, Matsui H, Rozenzhak S, Alakus H, Shepard PJ *et al*: **Genetic and epigenetic profiling of CLL disease progression reveals limited somatic evolution and suggests a relationship to memory-cell development**. *Blood Cancer J* 2015, **5**:e303.

16. Oakes CC, Seifert M, Assenov Y, Gu L, Przekopowitz M, Ruppert AS, Wang Q, Imbusch CD, Serva A, Koser SD *et al*: **DNA methylation dynamics during B cell maturation underlie a continuum of disease phenotypes in chronic lymphocytic leukemia**. *Nat Genet* 2016, **48**(3):253-264.

17. Papakonstantinou N, Ntoufa S, Tsagiopoulou M, Moysiadis T, Bhoi S, Malousi A, Psomopoulos F, Mansouri L, Laidou S, Papazoglou D *et al*: **Integrated epigenomic and transcriptomic analysis reveals TP63 as a novel player in clinically aggressive chronic lymphocytic leukemia**. *International journal of cancer* 2019, **144**(11):2695-2706.

18. Tsagiopoulou M, Papakonstantinou N, Moysiadis T, Mansouri L, Ljungström V, Duran-Ferrer M, Malousi A, Queirós AC, Plevova K, Bhoi S *et al*: **DNA methylation profiles in chronic lymphocytic leukemia patients treated with chemoimmunotherapy**. *Clinical epigenetics* 2019, **11**(1):177.

19. Barrow TM, Nakjang S, Lafta F, Bilotkach K, Woodhouse L, Junge G, Tudhope SJ, Wallis JP, Marr H, Marshall S *et al*: **Epigenome-wide analysis reveals functional modulators of drug sensitivity and post-treatment survival in chronic lymphocytic leukaemia**. *British journal of cancer* 2021, **124**(2):474-483.

20. Mansouri L, Wierzbinska JA, Plass C, Rosenquist R: **Epigenetic deregulation in chronic lymphocytic leukemia: Clinical and biological impact**. *Seminars in cancer biology* 2018, **51**:1-11.
