# Supplementary material for: Genome-wide DNA methylation profiling in chronic lymphocytic leukaemia
Source: Front Genet. 2023 Jan 11;13:1056043. doi: 10.3389/fgene.2022.1056043 (PMC9873975; doi:10.3389/fgene.2022.1056043)
Supplement: Supplementary file 11 [file Table2.doc]

Supplementary Table S2A. Public datasets used for methylation replication and transcription analysis in this study^a^

| Datasets^b^ | CLL | Healthy control |  |
| --- | --- | --- | --- |
| EGAD00010000254^(1)^ | 139 | 14^c^ | 450K DNA  methylation array |
| EGAD00010000871^(2)^ | 190^d^ | 7^e^ |
| EGAD00001000258^(3)^ | 97^e^ |  | RNA-seq |
| GSE66117^(4)^ | 47 |  |
| GSE62246^(5)^ |  | 2 |
| GSE70830^(6)^ |  | 5 |

^a^ For the consistency with our population, all samples of CLL patients and healthy subjects in public datasets involved in our study were CD19+ B cells.

^b^ Datasets of EGAD00010000254, EGAD00010000871, and EGAD00001000258 were requested from EGA. Datasets of GSE66117, GSE62246, and GSE70830 were downloaded from GEO.

^c^ 14 controls were selected in Supplementary Table S2B by selecting the “Description” of “DNA control” and the “Disease” of “B cell”.

^d^ 191 CLL cases of EGAD00010000871 were involved in Supplementary Table S2B by selecting the “Description” of “CLL cells derived from blood & CLL cells from blood” and “Disease” of “CLL”, and then we conducted the same process of quality control on methylation and removed one unqualified sample. So finally we included 190 samples of CLL cases for DMPs detection.

^e^ 7 control samples of EGAD00010000871 were involved in Table S2B by selecting the “Description” of “CD19+ sorted B cells from blood”.

^f^ RNA-sequencing data in CLL samples from 98 patients of EGAD00001000258 were in Supplementary Table S2C. We removed a sample for total count of genes less than 10,0000 before the differentially expressed analysis.

1. Kulis M, Heath S, Bibikova M, Queiros AC, Navarro A, Clot G, et al. Epigenomic analysis detects widespread gene-body DNA hypomethylation in chronic lymphocytic leukemia. Nat Genet. 2012;44(11):1236-42.

2. Oakes CC, Claus R, Gu L, Assenov Y, Hullein J, Zucknick M, et al. Evolution of DNA methylation is linked to genetic aberrations in chronic lymphocytic leukemia. Cancer Discov. 2014;4(3):348-61.

3. Ferreira PG, Jares P, Rico D, Gomez-Lopez G, Martinez-Trillos A, Villamor N, et al. Transcriptome characterization by RNA sequencing identifies a major molecular and clinical subdivision in chronic lymphocytic leukemia. Genome Res. 2014;24(2):212-26.

4. Kushwaha G, Dozmorov M, Wren JD, Qiu J, Shi H, Xu D. Hypomethylation coordinates antagonistically with hypermethylation in cancer development: a case study of leukemia. Hum Genomics. 2016;10 Suppl 2:18.

5. Koues OI, Kowalewski RA, Chang LW, Pyfrom SC, Schmidt JA, Luo H, et al. Enhancer sequence variants and transcription-factor deregulation synergize to construct pathogenic regulatory circuits in B-cell lymphoma. Immunity. 2015;42(1):186-98.

6. Liao W, Jordaan G, Nham P, Phan RT, Pelegrini M, Sharma S. Gene expression and splicing alterations analyzed by high throughput RNA sequencing of chronic lymphocytic leukemia specimens. BMC Cancer. 2015;15:714.

Supplementary Table S2B. Clinical characteristics of samples used for methylation replication (EGAD00010000871 and EGAD00010000254)

|  | Sample | Source | Description | Organism | Sex | Disease | Sentrix_ID | Sentrix_Position | Study |
| --- | --- | --- | --- | --- | --- | --- | --- | --- | --- |
| 1 | CB_D1 | E-T-1 | B cells sorted from tonsil | tonsil | Male | Normal | 9829119254 | R05C01 | EGAD00010000871 |
| 2 | CB_D2 | E-T-2 | B cells sorted from tonsil | tonsil | Male | Normal | 9878820137 | R01C01 | EGAD00010000871 |
| 3 | CB_D3 | E-T-3 | B cells sorted from tonsil | tonsil | Female | Normal | 9878820137 | R03C01 | EGAD00010000871 |
| 4 | CC_D1 | E-T-1 | B cells sorted from tonsil | tonsil | Male | Normal | 9829119254 | R06C01 | EGAD00010000871 |
| 5 | CC_D2 | E-T-2 | B cells sorted from tonsil | tonsil | Male | Normal | 9878820137 | R02C01 | EGAD00010000871 |
| 6 | CC_D3 | E-T-3 | B cells sorted from tonsil | tonsil | Female | Normal | 9878820137 | R04C01 | EGAD00010000871 |
| 7 | CLL100_2010 | CLL100 | CLL cells derived from blood | PBMC | Female | CLL | 8363800197 | R04C02 | EGAD00010000871 |
| 8 | CLL100_2011 | CLL100 | CLL cells derived from blood | PBMC | Female | CLL | 8622007056 | R05C02 | EGAD00010000871 |
| 9 | CLL101 | CLL101 | CLL cells derived from blood | PBMC | Male | CLL | 8363800198 | R05C01 | EGAD00010000871 |
| 10 | CLL102 | CLL102 | CLL cells derived from blood | PBMC | Male | CLL | 8363800196 | R02C02 | EGAD00010000871 |
| 11 | CLL103 | CLL103 | CLL cells derived from blood | PBMC | Male | CLL | 6285641017 | R02C01 | EGAD00010000871 |
| 12 | CLL104 | CLL104 | CLL cells derived from blood | PBMC | Male | CLL | 8363800196 | R02C01 | EGAD00010000871 |
| 13 | CLL105 | CLL105 | CLL cells derived from blood | PBMC | Female | CLL | 8363800197 | R06C02 | EGAD00010000871 |
| 14 | CLL106 | CLL106 | CLL cells derived from blood | PBMC | Female | CLL | 6285641017 | R03C01 | EGAD00010000871 |
| 15 | CLL107 | CLL107 | CLL cells derived from blood | PBMC | Female | CLL | 8363800197 | R06C01 | EGAD00010000871 |
| 16 | CLL108 | CLL108 | CLL cells derived from blood | PBMC | Male | CLL | 8363800196 | R06C01 | EGAD00010000871 |
| 17 | CLL109 | CLL109 | CLL cells derived from blood | PBMC | Female | CLL | 8363800196 | R05C02 | EGAD00010000871 |
| 18 | CLL110 | CLL110 | CLL cells derived from blood | PBMC | Female | CLL | 8363800196 | R06C02 | EGAD00010000871 |
| 19 | CLL111 | CLL111 | CLL cells derived from blood | PBMC | Female | CLL | 8363800197 | R01C01 | EGAD00010000871 |
| 20 | CLL112 | CLL112 | CLL cells derived from blood | PBMC | Female | CLL | 6285641017 | R02C02 | EGAD00010000871 |
| 21 | CLL113 | CLL113 | CLL cells derived from blood | PBMC | Male | CLL | 6285641017 | R06C01 | EGAD00010000871 |
| 22 | CLL114 | CLL114 | CLL cells derived from blood | PBMC | Male | CLL | 8363800197 | R03C01 | EGAD00010000871 |
| 23 | CLL115 | CLL115 | CLL cells derived from blood | PBMC | Male | CLL | 8363800197 | R05C01 | EGAD00010000871 |
| 24 | CLL116 | CLL116 | CLL cells derived from blood | PBMC | Female | CLL | 8363800197 | R01C02 | EGAD00010000871 |
| 25 | CLL117 | CLL117 | CLL cells derived from blood | PBMC | Male | CLL | 8363800197 | R02C02 | EGAD00010000871 |
| 26 | CLL118 | CLL118 | CLL cells derived from blood | PBMC | Male | CLL | 6285641017 | R05C01 | EGAD00010000871 |
| 27 | CLL119 | CLL119 | CLL cells derived from blood | PBMC | Female | CLL | 8363800197 | R03C02 | EGAD00010000871 |
| 28 | CLL120 | CLL120 | CLL cells derived from blood | PBMC | Female | CLL | 8363800196 | R04C02 | EGAD00010000871 |
| 29 | CLL121 | CLL121 | CLL cells derived from blood | PBMC | Male | CLL | 6285641017 | R04C01 | EGAD00010000871 |
| 30 | CLL122 | CLL122 | CLL cells derived from blood | PBMC | Male | CLL | 8363800197 | R05C02 | EGAD00010000871 |
| 31 | CLL123 | CLL123 | CLL cells derived from blood | PBMC | Female | CLL | 9003865025 | R01C01 | EGAD00010000871 |
| 32 | CLL124 | CLL124 | CLL cells derived from blood | PBMC | Male | CLL | 9340996140 | R01C01 | EGAD00010000871 |
| 33 | CLL125 | CLL125 | CLL cells derived from blood | PBMC | Female | CLL | 9340996140 | R01C02 | EGAD00010000871 |
| 34 | CLL126 | CLL126 | CLL cells derived from blood | PBMC | Male | CLL | 9003865025 | R02C01 | EGAD00010000871 |
| 35 | CLL127 | CLL127 | CLL cells derived from blood | PBMC | Male | CLL | 9305216173 | R02C01 | EGAD00010000871 |
| 36 | CLL128 | CLL128 | CLL cells derived from blood | PBMC | Male | CLL | 9340996140 | R02C01 | EGAD00010000871 |
| 37 | CLL129 | CLL129 | CLL cells derived from blood | PBMC | Female | CLL | 9305216173 | R02C02 | EGAD00010000871 |
| 38 | CLL130 | CLL130 | CLL cells derived from blood | PBMC | Female | CLL | 9340996140 | R02C02 | EGAD00010000871 |
| 39 | CLL131 | CLL131 | CLL cells derived from blood | PBMC | Female | CLL | 9003865025 | R03C01 | EGAD00010000871 |
| 40 | CLL132 | CLL132 | CLL cells derived from blood | PBMC | Male | CLL | 9340996140 | R03C01 | EGAD00010000871 |
| 41 | CLL133 | CLL133 | CLL cells derived from blood | PBMC | Male | CLL | 9340996140 | R03C02 | EGAD00010000871 |
| 42 | CLL134 | CLL134 | CLL cells derived from blood | PBMC | Male | CLL | 9003865025 | R04C01 | EGAD00010000871 |
| 43 | CLL135 | CLL135 | CLL cells derived from blood | PBMC | Male | CLL | 9305216173 | R04C01 | EGAD00010000871 |
| 44 | CLL136 | CLL136 | CLL cells derived from blood | PBMC | Male | CLL | 9340996140 | R04C01 | EGAD00010000871 |
| 45 | CLL137 | CLL137 | CLL cells derived from blood | PBMC | Female | CLL | 9305216173 | R04C02 | EGAD00010000871 |
| 46 | CLL138 | CLL138 | CLL cells derived from blood | PBMC | Male | CLL | 9340996140 | R04C02 | EGAD00010000871 |
| 47 | CLL139 | CLL139 | CLL cells derived from blood | PBMC | Male | CLL | 9340996140 | R05C01 | EGAD00010000871 |
| 48 | CLL140 | CLL140 | CLL cells derived from blood | PBMC | Female | CLL | 9340996140 | R05C02 | EGAD00010000871 |
| 49 | CLL141 | CLL141 | CLL cells derived from blood | PBMC | Male | CLL | 9305216173 | R06C01 | EGAD00010000871 |
| 50 | CLL142 | CLL142 | CLL cells derived from blood | PBMC | Male | CLL | 9340996140 | R06C01 | EGAD00010000871 |
| 51 | CLL143 | CLL143 | CLL cells derived from blood | PBMC | Male | CLL | 9376538165 | R01C02 | EGAD00010000871 |
| 52 | CLL144 | CLL144 | CLL cells derived from blood | PBMC | Male | CLL | 9376538165 | R02C02 | EGAD00010000871 |
| 53 | CLL145 | CLL145 | CLL cells derived from blood | PBMC | Female | CLL | 9376538165 | R03C01 | EGAD00010000871 |
| 54 | CLL146 | CLL146 | CLL cells derived from blood | PBMC | Male | CLL | 9376538165 | R03C02 | EGAD00010000871 |
| 55 | CLL147 | CLL147 | CLL cells derived from blood | PBMC | Female | CLL | 9376538165 | R04C01 | EGAD00010000871 |
| 56 | CLL148 | CLL148 | CLL cells derived from blood | PBMC | Male | CLL | 9376538165 | R04C02 | EGAD00010000871 |
| 57 | CLL149 | CLL149 | CLL cells derived from blood | PBMC | Female | CLL | 9376538165 | R05C01 | EGAD00010000871 |
| 58 | CLL150 | CLL150 | CLL cells derived from blood | PBMC | Female | CLL | 9376538165 | R05C02 | EGAD00010000871 |
| 59 | CLL151 | CLL151 | CLL cells derived from blood | PBMC | Female | CLL | 9376538165 | R06C01 | EGAD00010000871 |
| 60 | CLL152 | CLL152 | CLL cells derived from blood | PBMC | Male | CLL | 9376538165 | R06C02 | EGAD00010000871 |
| 61 | CLL153 | CLL153 | CLL cells derived from blood | PBMC | Male | CLL | 9422493122 | R01C01 | EGAD00010000871 |
| 62 | CLL154 | CLL154 | CLL cells derived from blood | PBMC | Male | CLL | 9422493122 | R02C01 | EGAD00010000871 |
| 63 | CLL155 | CLL155 | CLL cells derived from blood | PBMC | Male | CLL | 9422493122 | R03C01 | EGAD00010000871 |
| 64 | CLL156 | CLL156 | CLL cells derived from blood | PBMC | Male | CLL | 9422493122 | R04C01 | EGAD00010000871 |
| 65 | CLL157 | CLL157 | CLL cells derived from blood | PBMC | Male | CLL | 9422493114 | R04C02 | EGAD00010000871 |
| 66 | CLL158 | CLL158 | CLL cells derived from blood | PBMC | Male | CLL | 9422493114 | R05C02 | EGAD00010000871 |
| 67 | CLL159 | CLL159 | CLL cells derived from blood | PBMC | Male | CLL | 9969477095 | R06C01 | EGAD00010000871 |
| 68 | CLL160 | CLL160 | CLL cells derived from blood | PBMC | Male | CLL | 9969477095 | R01C02 | EGAD00010000871 |
| 69 | CLL161 | CLL161 | CLL cells derived from blood | PBMC | Female | CLL | 9969477095 | R02C02 | EGAD00010000871 |
| 70 | CLL162 | CLL162 | CLL cells derived from blood | PBMC | Male | CLL | 9969477095 | R03C02 | EGAD00010000871 |
| 71 | CLL163 | CLL163 | CLL cells derived from blood | PBMC | Male | CLL | 9969477095 | R04C02 | EGAD00010000871 |
| 72 | CLL164 | CLL164 | CLL cells derived from blood | PBMC | Male | CLL | 9969477095 | R06C02 | EGAD00010000871 |
| 73 | CLL165 | CLL165 | CLL cells derived from blood | PBMC | Male | CLL | 9878820129 | R01C01 | EGAD00010000871 |
| 74 | CLL166 | CLL166 | CLL cells derived from blood | PBMC | Female | CLL | 9878820129 | R02C01 | EGAD00010000871 |
| 75 | CLL167 | CLL167 | CLL cells derived from blood | PBMC | Female | CLL | 9878820129 | R03C01 | EGAD00010000871 |
| 76 | CLL168 | CLL168 | CLL cells derived from blood | PBMC | Female | CLL | 9878820129 | R04C01 | EGAD00010000871 |
| 77 | CLL169 | CLL169 | CLL cells derived from blood | PBMC | Male | CLL | 9878820129 | R05C01 | EGAD00010000871 |
| 78 | CLL170 | CLL170 | CLL cells derived from blood | PBMC | Female | CLL | 9878820129 | R06C01 | EGAD00010000871 |
| 79 | CLL171 | CLL171 | CLL cells derived from blood | PBMC | Female | CLL | 9878820129 | R01C02 | EGAD00010000871 |
| 80 | CLL172 | CLL172 | CLL cells derived from blood | PBMC | Male | CLL | 9878820129 | R02C02 | EGAD00010000871 |
| 81 | CLL173 | CLL173 | CLL cells derived from blood | PBMC | Female | CLL | 9878820129 | R03C02 | EGAD00010000871 |
| 82 | CLL174 | CLL174 | CLL cells derived from blood | PBMC | Male | CLL | 9878820129 | R04C02 | EGAD00010000871 |
| 83 | CLL175 | CLL175 | CLL cells derived from blood | PBMC | Female | CLL | 9878820129 | R05C02 | EGAD00010000871 |
| 84 | CLL176 | CLL176 | CLL cells derived from blood | PBMC | Male | CLL | 9878820129 | R06C02 | EGAD00010000871 |
| 85 | CLL177 | CLL177 | CLL cells derived from blood | PBMC | Female | CLL | 9829119165 | R01C01 | EGAD00010000871 |
| 86 | CLL178 | CLL178 | CLL cells derived from blood | PBMC | Female | CLL | 9829119165 | R02C01 | EGAD00010000871 |
| 87 | CLL179 | CLL179 | CLL cells derived from blood | PBMC | Male | CLL | 9829119165 | R03C01 | EGAD00010000871 |
| 88 | CLL180 | CLL180 | CLL cells from blood | PBMC | Female | CLL | 9829119165 | R04C01 | EGAD00010000871 |
| 89 | CLL181 | CLL181 | CLL cells from blood | PBMC | Male | CLL | 9829119165 | R05C01 | EGAD00010000871 |
| 90 | CLL182 | CLL182 | CLL cells from blood | PBMC | Male | CLL | 9829119165 | R06C01 | EGAD00010000871 |
| 91 | CLL183 | CLL183 | CLL cells from blood | PBMC | Male | CLL | 9829119165 | R01C02 | EGAD00010000871 |
| 92 | CLL184 | CLL184 | CLL cells from blood | PBMC | Female | CLL | 9829119165 | R02C02 | EGAD00010000871 |
| 93 | CLL185 | CLL185 | CLL cells from blood | PBMC | Female | CLL | 9829119165 | R03C02 | EGAD00010000871 |
| 94 | CLL186 | CLL186 | CLL cells from blood | PBMC | Female | CLL | 9829119165 | R04C02 | EGAD00010000871 |
| 95 | CLL187 | CLL187 | CLL cells from blood | PBMC | Male | CLL | 9829119165 | R05C02 | EGAD00010000871 |
| 96 | CLL188 | CLL188 | CLL cells from blood | PBMC | Male | CLL | 9829119165 | R06C02 | EGAD00010000871 |
| 97 | CLL189 | CLL189 | CLL cells from blood | PBMC | Female | CLL | 9829119254 | R05C02 | EGAD00010000871 |
| 98 | CLL190 | CLL190 | CLL cells from blood | PBMC | Male | CLL | 9829119254 | R06C02 | EGAD00010000871 |
| 99 | CLL191 | CLL191 | CLL cells from blood | PBMC | Male | CLL | 9878820137 | R01C02 | EGAD00010000871 |
| 100 | CLL192 | CLL192 | CLL cells from blood | PBMC | Female | CLL | 9878820137 | R02C02 | EGAD00010000871 |
| 101 | CLL193 | CLL193 | CLL cells from blood | PBMC | Male | CLL | 9878820137 | R03C02 | EGAD00010000871 |
| 102 | CLL194 | CLL194 | CLL cells from blood | PBMC | Female | CLL | 9878820117 | R01C01 | EGAD00010000871 |
| 103 | CLL195 | CLL195 | CLL cells from blood | PBMC | Male | CLL | 9878820117 | R02C01 | EGAD00010000871 |
| 104 | CLL196 | CLL196 | CLL cells from blood | PBMC | Male | CLL | 9878820117 | R03C01 | EGAD00010000871 |
| 105 | CLL197 | CLL197 | CLL cells from blood | PBMC | Male | CLL | 9878820117 | R04C01 | EGAD00010000871 |
| 106 | CLL198 | CLL198 | CLL cells from blood | PBMC | Female | CLL | 9878820117 | R05C01 | EGAD00010000871 |
| 107 | CLL199 | CLL199 | CLL cells from blood | PBMC | Female | CLL | 9878820117 | R06C01 | EGAD00010000871 |
| 108 | CLL20 | CLL20 | CLL cells from blood | PBMC | Female | CLL | 9968646148 | R01C02 | EGAD00010000871 |
| 109 | CLL200 | CLL200 | CLL cells from blood | PBMC | Female | CLL | 9878820117 | R01C02 | EGAD00010000871 |
| 110 | CLL21 | CLL21 | CLL cells from blood | PBMC | Male | CLL | 6969568043 | R01C01 | EGAD00010000871 |
| 111 | CLL23 | CLL23 | CLL cells from blood | PBMC | Male | CLL | 6969568043 | R02C01 | EGAD00010000871 |
| 112 | CLL26 | CLL26 | CLL cells from blood | PBMC | Male | CLL | 9533774069 | R01C01 | EGAD00010000871 |
| 113 | CLL30 | CLL30 | CLL cells from blood | PBMC | Male | CLL | 6969568043 | R03C01 | EGAD00010000871 |
| 114 | CLL31 | CLL31 | CLL cells from blood | PBMC | Male | CLL | 6969568043 | R04C01 | EGAD00010000871 |
| 115 | CLL32_2007 | CLL32 | CLL cells from blood | PBMC | Male | CLL | 8363800244 | R06C01 | EGAD00010000871 |
| 116 | CLL32_2011 | CLL32 | CLL cells from blood | PBMC | Male | CLL | 6969568043 | R05C01 | EGAD00010000871 |
| 117 | CLL33 | CLL33 | CLL cells from blood | PBMC | Female | CLL | 6969568043 | R06C01 | EGAD00010000871 |
| 118 | CLL34 | CLL34 | CLL cells from blood | PBMC | Male | CLL | 6969568043 | R01C02 | EGAD00010000871 |
| 119 | CLL35 | CLL35 | CLL cells from blood | PBMC | Male | CLL | 6969568043 | R02C02 | EGAD00010000871 |
| 120 | CLL36_2002 | CLL36 | CLL cells from blood | PBMC | Male | CLL | 8363800244 | R05C01 | EGAD00010000871 |
| 121 | CLL36_2011 | CLL36 | CLL cells from blood | PBMC | Male | CLL | 6969568043 | R03C02 | EGAD00010000871 |
| 122 | CLL37 | CLL37 | CLL cells from blood | PBMC | Female | CLL | 9533774069 | R02C01 | EGAD00010000871 |
| 123 | CLL39 | CLL39 | CLL cells from blood | PBMC | Male | CLL | 9533774069 | R03C01 | EGAD00010000871 |
| 124 | CLL40 | CLL40 | CLL cells from blood | PBMC | Female | CLL | 6969568043 | R04C02 | EGAD00010000871 |
| 125 | CLL42 | CLL42 | CLL cells from blood | PBMC | Male | CLL | 6969568043 | R05C02 | EGAD00010000871 |
| 126 | CLL43 | CLL43 | CLL cells from blood | PBMC | Male | CLL | 6969568043 | R06C02 | EGAD00010000871 |
| 127 | CLL44 | CLL44 | CLL cells from blood | PBMC | Female | CLL | 6969568044 | R01C01 | EGAD00010000871 |
| 128 | CLL45 | CLL45 | CLL cells from blood | PBMC | Male | CLL | 6969568044 | R02C01 | EGAD00010000871 |
| 129 | CLL46 | CLL46 | CLL cells from blood | PBMC | Female | CLL | 9533774069 | R04C01 | EGAD00010000871 |
| 130 | CLL47 | CLL47 | CLL cells from blood | PBMC | Male | CLL | 8363800244 | R03C02 | EGAD00010000871 |
| 131 | CLL48_2010 | CLL48 | CLL cells from blood | PBMC | Male | CLL | 8363800197 | R04C01 | EGAD00010000871 |
| 132 | CLL48_2012 | CLL48 | CLL cells from blood | PBMC | Male | CLL | 8622007057 | R01C01 | EGAD00010000871 |
| 133 | CLL49 | CLL49 | CLL cells from blood | PBMC | Male | CLL | 9533774069 | R05C01 | EGAD00010000871 |
| 134 | CLL51 | CLL51 | CLL cells from blood | PBMC | Female | CLL | 6969568044 | R03C01 | EGAD00010000871 |
| 135 | CLL52 | CLL52 | CLL cells from blood | PBMC | Male | CLL | 9422493122 | R05C01 | EGAD00010000871 |
| 136 | CLL53 | CLL53 | CLL cells from blood | PBMC | Male | CLL | 9422493122 | R06C01 | EGAD00010000871 |
| 137 | CLL54 | CLL54 | CLL cells from blood | PBMC | Female | CLL | 9533774069 | R06C01 | EGAD00010000871 |
| 138 | CLL55 | CLL55 | CLL cells from blood | PBMC | Male | CLL | 9533774069 | R01C02 | EGAD00010000871 |
| 139 | CLL57 | CLL57 | CLL cells from blood | PBMC | Female | CLL | 9533774069 | R02C02 | EGAD00010000871 |
| 140 | CLL58 | CLL58 | CLL cells from blood | PBMC | Male | CLL | 9968646148 | R02C02 | EGAD00010000871 |
| 141 | CLL59 | CLL59 | CLL cells from blood | PBMC | Male | CLL | 9533774069 | R03C02 | EGAD00010000871 |
| 142 | CLL60 | CLL60 | CLL cells from blood | PBMC | Male | CLL | 6285641016 | R05C02 | EGAD00010000871 |
| 143 | CLL62_2002 | CLL62 | CLL cells from blood | PBMC | Female | CLL | 8363800244 | R01C02 | EGAD00010000871 |
| 144 | CLL62_2012 | CLL62 | CLL cells from blood | PBMC | Female | CLL | 6285641016 | R06C02 | EGAD00010000871 |
| 145 | CLL63_2008 | CLL63 | CLL cells from blood | PBMC | Female | CLL | 8363800244 | R02C02 | EGAD00010000871 |
| 146 | CLL63_2012 | CLL63 | CLL cells from blood | PBMC | Xo | CLL | 8622007057 | R01C02 | EGAD00010000871 |
| 147 | CLL64 | CLL64 | CLL cells from blood | PBMC | Male | CLL | 9533774069 | R04C02 | EGAD00010000871 |
| 148 | CLL65_2010 | CLL65 | CLL cells from blood | PBMC | Female | CLL | 8622007057 | R03C01 | EGAD00010000871 |
| 149 | CLL65_2012 | CLL65 | CLL cells from blood | PBMC | Female | CLL | 8622007057 | R04C01 | EGAD00010000871 |
| 150 | CLL66 | CLL66 | CLL cells from blood | PBMC | Male | CLL | 9968646148 | R03C02 | EGAD00010000871 |
| 151 | CLL68 | CLL68 | CLL cells from blood | PBMC | Male | CLL | 9553932009 | R06C02 | EGAD00010000871 |
| 152 | CLL71 | CLL71 | CLL cells from blood | PBMC | Female | CLL | 9968646148 | R04C02 | EGAD00010000871 |
| 153 | CLL72 | CLL72 | CLL cells from blood | PBMC | Male | CLL | 6285641017 | R01C01 | EGAD00010000871 |
| 154 | CLL73_2010 | CLL73 | CLL cells from blood | PBMC | Male | CLL | 8363800196 | R01C02 | EGAD00010000871 |
| 155 | CLL73_2012 | CLL73 | CLL cells from blood | PBMC | Male | CLL | 8622007057 | R02C01 | EGAD00010000871 |
| 156 | CLL77 | CLL77 | CLL cells from blood | PBMC | Female | CLL | 9968646148 | R06C02 | EGAD00010000871 |
| 157 | CLL79_2010 | CLL79 | CLL cells from blood | PBMC | Female | CLL | 8363800196 | R03C02 | EGAD00010000871 |
| 158 | CLL79_2011 | CLL79 | CLL cells from blood | PBMC | Female | CLL | 8622007056 | R06C02 | EGAD00010000871 |
| 159 | CLL80_2010 | CLL80 | CLL cells from blood | PBMC | Male | CLL | 8363800196 | R03C01 | EGAD00010000871 |
| 160 | CLL80_2011 | CLL80 | CLL cells from blood | PBMC | Male | CLL | 8622007056 | R01C02 | EGAD00010000871 |
| 161 | CLL81_2010 | CLL81 | CLL cells from blood | PBMC | Female | CLL | 8363800196 | R04C01 | EGAD00010000871 |
| 162 | CLL81_2012 | CLL81 | CLL cells from blood | PBMC | Female | CLL | 8622007056 | R03C02 | EGAD00010000871 |
| 163 | CLL82_2003 | CLL82 | CLL cells from blood | PBMC | Male | CLL | 8363800198 | R05C02 | EGAD00010000871 |
| 164 | CLL82_2004 | CLL82 | CLL cells from blood | PBMC | Male | CLL | 8363800198 | R06C02 | EGAD00010000871 |
| 165 | CLL83_2010 | CLL83 | CLL cells from blood | PBMC | Female | CLL | 8622007057 | R05C01 | EGAD00010000871 |
| 166 | CLL83_2012 | CLL83 | CLL cells from blood | PBMC | Female | CLL | 8622007057 | R06C01 | EGAD00010000871 |
| 167 | CLL84_2004 | CLL84 | CLL cells from blood | PBMC | Male | CLL | 8363800244 | R05C02 | EGAD00010000871 |
| 168 | CLL84_2008 | CLL84 | CLL cells from blood | PBMC | Male | CLL | 8363800244 | R06C02 | EGAD00010000871 |
| 169 | CLL85_2003 | CLL85 | CLL cells from blood | PBMC | Male | CLL | 8622007056 | R04C01 | EGAD00010000871 |
| 170 | CLL85_2009 | CLL85 | CLL cells from blood | PBMC | Male | CLL | 8622007056 | R05C01 | EGAD00010000871 |
| 171 | CLL86_2006 | CLL86 | CLL cells from blood | PBMC | Female | CLL | 6285641016 | R03C01 | EGAD00010000871 |
| 172 | CLL86_2011 | CLL86 | CLL cells from blood | PBMC | Female | CLL | 8363800244 | R04C02 | EGAD00010000871 |
| 173 | CLL87_2007 | CLL87 | CLL cells from blood | PBMC | Male | CLL | 8622007056 | R01C01 | EGAD00010000871 |
| 174 | CLL87_2009 | CLL87 | CLL cells from blood | PBMC | Male | CLL | 8622007056 | R03C01 | EGAD00010000871 |
| 175 | CLL88_2009 | CLL88 | CLL cells from blood | PBMC | Male | CLL | 6285641016 | R01C02 | EGAD00010000871 |
| 176 | CLL88_2010 | CLL88 | CLL cells from blood | PBMC | Male | CLL | 6285641016 | R02C02 | EGAD00010000871 |
| 177 | CLL89_2010 | CLL89 | CLL cells from blood | PBMC | Male | CLL | 8363800197 | R02C01 | EGAD00010000871 |
| 178 | CLL89_2011 | CLL89 | CLL cells from blood | PBMC | Male | CLL | 8622007056 | R04C02 | EGAD00010000871 |
| 179 | CLL90_2003 | CLL90 | CLL cells from blood | PBMC | Female | CLL | 8363800198 | R03C02 | EGAD00010000871 |
| 180 | CLL90_2009 | CLL90 | CLL cells from blood | PBMC | Female | CLL | 8363800198 | R04C02 | EGAD00010000871 |
| 181 | CLL91_2002 | CLL91 | CLL cells from blood | PBMC | Male | CLL | 6285641016 | R05C01 | EGAD00010000871 |
| 182 | CLL91_2008 | CLL91 | CLL cells from blood | PBMC | Male | CLL | 6285641016 | R06C01 | EGAD00010000871 |
| 183 | CLL92_2003 | CLL92 | CLL cells from blood | PBMC | Male | CLL | 6285641016 | R01C01 | EGAD00010000871 |
| 184 | CLL92_2007 | CLL92 | CLL cells from blood | PBMC | Male | CLL | 6285641016 | R02C01 | EGAD00010000871 |
| 185 | CLL93_2004 | CLL93 | CLL cells from blood | PBMC | Female | CLL | 8363800198 | R01C01 | EGAD00010000871 |
| 186 | CLL93_2005 | CLL93 | CLL cells from blood | PBMC | Female | CLL | 8363800198 | R02C01 | EGAD00010000871 |
| 187 | CLL94_2010 | CLL94 | CLL cells from blood | PBMC | Female | CLL | 8363800196 | R01C01 | EGAD00010000871 |
| 188 | CLL94_2011 | CLL94 | CLL cells from blood | PBMC | Female | CLL | 8622007056 | R06C01 | EGAD00010000871 |
| 189 | CLL95_2004 | CLL95 | CLL cells from blood | PBMC | Male | CLL | 8363800198 | R03C01 | EGAD00010000871 |
| 190 | CLL95_2005 | CLL95 | CLL cells from blood | PBMC | Male | CLL | 8363800198 | R04C01 | EGAD00010000871 |
| 191 | CLL96_2002 | CLL96 | CLL cells from blood | PBMC | Male | CLL | 6285641016 | R03C02 | EGAD00010000871 |
| 192 | CLL96_2005 | CLL96 | CLL cells from blood | PBMC | Male | CLL | 6285641016 | R04C02 | EGAD00010000871 |
| 193 | CLL97_2005 | CLL97 | CLL cells from blood | PBMC | Female | CLL | 8363800198 | R01C02 | EGAD00010000871 |
| 194 | CLL97_2010 | CLL97 | CLL cells from blood | PBMC | Female | CLL | 8363800198 | R02C02 | EGAD00010000871 |
| 195 | CLL98 | CLL98 | CLL cells from blood | PBMC | Male | CLL | 6285641017 | R01C02 | EGAD00010000871 |
| 196 | CLL99_2010 | CLL99 | CLL cells from blood | PBMC | Male | CLL | 8363800196 | R05C01 | EGAD00010000871 |
| 197 | CLL99_2011 | CLL99 | CLL cells from blood | PBMC | Male | CLL | 8622007056 | R02C02 | EGAD00010000871 |
| 198 | GCF_D5 | E-T-5 | B cells sorted from tonsil | tonsil | Female | Normal | 9968646144 | R05C02 | EGAD00010000871 |
| 199 | GCF_D6 | E-T-6 | B cells sorted from tonsil | tonsil | Female | Normal | 9534104046 | R06C01 | EGAD00010000871 |
| 200 | GCF_D7 | E-T-7 | B cells sorted from tonsil | tonsil | Male | Normal | 9878820137 | R05C01 | EGAD00010000871 |
| 201 | HB15 | G-B-15 | CD19+ sorted B cells from blood | blood | Female | Normal | 6969568044 | R01C02 | EGAD00010000871 |
| 202 | HB18 | G-B-18 | CD19+ sorted B cells from blood | blood | Female | Normal | 6969568044 | R02C02 | EGAD00010000871 |
| 203 | HB3 | G-B-3 | CD19+ sorted B cells from blood | blood | Male | Normal | 6969568044 | R04C01 | EGAD00010000871 |
| 204 | HB45 | U-B-45 | CD19+ sorted B cells from blood | blood | Male | Normal | 8622007057 | R02C02 | EGAD00010000871 |
| 205 | HB48 | U-B-48 | CD19+ sorted B cells from blood | blood | Male | Normal | 8622007057 | R03C02 | EGAD00010000871 |
| 206 | HB7 | G-B-7 | CD19+ sorted B cells from blood | blood | Male | Normal | 6969568044 | R05C01 | EGAD00010000871 |
| 207 | HB8 | G-B-8 | CD19+ sorted B cells from blood | blood | Female | Normal | 6969568044 | R06C01 | EGAD00010000871 |
| 208 | hiMBC_D5 | E-B-5 | B cells sorted from blood | blood | Male | Normal | 9553932009 | R02C02 | EGAD00010000871 |
| 209 | hiMBC_D7 | E-B-7 | B cells sorted from blood | blood | Male | Normal | 9553932009 | R05C02 | EGAD00010000871 |
| 210 | HT1 | G-B-1 | CD3+ sorted T cells from blood | blood | Male | Normal | 6969568044 | R04C02 | EGAD00010000871 |
| 211 | HT17 | G-B-17 | CD3+ sorted T cells from blood | blood | Male | Normal | 6969568044 | R03C02 | EGAD00010000871 |
| 212 | HT2 | G-B-2 | CD3+ sorted T cells from blood | blood | Female | Normal | 6969568044 | R05C02 | EGAD00010000871 |
| 213 | HT3 | G-B-2 | CD3+ sorted T cells from blood | blood | Female | Normal | 6969568044 | R06C02 | EGAD00010000871 |
| 214 | intMBC_D1 | E-B-1 | B cells sorted from blood | blood | Female | Normal | 9829119254 | R01C01 | EGAD00010000871 |
| 215 | intMBC_D2 | E-B-2 | B cells sorted from blood | blood | Female | Normal | 9829119254 | R02C01 | EGAD00010000871 |
| 216 | intMBC_D4 | E-B-4 | B cells sorted from blood | blood | Female | Normal | 9829119254 | R04C01 | EGAD00010000871 |
| 217 | loMBC_D10 | E-B-10 | B cells sorted from blood | blood | Female | Normal | 9534104046 | R02C02 | EGAD00010000871 |
| 218 | loMBC_D5 | E-B-3 | B cells sorted from blood | blood | Male | Normal | 9553932009 | R01C02 | EGAD00010000871 |
| 219 | loMBC_D7 | E-B-7 | B cells sorted from blood | blood | Male | Normal | 9553932009 | R04C02 | EGAD00010000871 |
| 220 | loMBC_D9 | E-B-9 | B cells sorted from blood | blood | Male | Normal | 9534104046 | R01C02 | EGAD00010000871 |
| 221 | MGZ_D1 | E-S-1 | B cells sorted from spleen | spleen | Female | Normal | 9534104046 | R03C01 | EGAD00010000871 |
| 222 | MGZ_D2 | E-S-2 | B cells sorted from spleen | spleen | Female | Normal | 9534104046 | R04C01 | EGAD00010000871 |
| 223 | MGZ_D3 | E-S-3 | B cells sorted from spleen | spleen | Male | Normal | 9534104046 | R05C01 | EGAD00010000871 |
| 224 | NBC_D10 | E-B-10 | B cells sorted from blood | blood | Female | Normal | 9969477033 | R02C01 | EGAD00010000871 |
| 225 | NBC_D5 | E-B-5 | B cells sorted from blood | blood | Male | Normal | 9553932009 | R06C01 | EGAD00010000871 |
| 226 | NBC_D7 | E-B-7 | B cells sorted from blood | blood | Male | Normal | 9553932009 | R03C02 | EGAD00010000871 |
| 227 | 001-0002-11TD | NA | DNA CLL | NA | Male | CLL | 5822986021 | R01C01 | EGAD00010000254 |
| 228 | 002-0010-10TD | NA | DNA CLL | NA | Female | CLL | 5823031009 | R01C01 | EGAD00010000254 |
| 229 | 003-0005-10TD | NA | DNA CLL | NA | Male | CLL | 5823031011 | R01C01 | EGAD00010000254 |
| 230 | 004-0012-05TD | NA | DNA CLL | NA | Female | CLL | 5823031021 | R01C01 | EGAD00010000254 |
| 231 | 005-0015-01TD | NA | DNA CLL | NA | Male | CLL | 5854945025 | R01C01 | EGAD00010000254 |
| 232 | 007-0020-01TD | NA | DNA CLL | NA | Male | CLL | 5854945037 | R01C01 | EGAD00010000254 |
| 233 | 008-0022-01TD | NA | DNA CLL | NA | Male | CLL | 5854945039 | R01C01 | EGAD00010000254 |
| 234 | 009-0026-02TD | NA | DNA CLL | NA | Male | CLL | 5854945059 | R01C01 | EGAD00010000254 |
| 235 | 010-0028-02TD | NA | DNA CLL | NA | Male | CLL | 5822986021 | R02C01 | EGAD00010000254 |
| 236 | 012-02-1TD | NA | DNA CLL | NA | Female | CLL | 5823031009 | R02C01 | EGAD00010000254 |
| 237 | 014-0037-02TD | NA | DNA CLL | NA | Female | CLL | 5823031011 | R02C01 | EGAD00010000254 |
| 238 | 015-0038-01TD | NA | DNA CLL | NA | Male | CLL | 5823031021 | R02C01 | EGAD00010000254 |
| 239 | 016-0040-02TD | NA | DNA CLL | NA | Male | CLL | 5854945025 | R02C01 | EGAD00010000254 |
| 240 | 017-0042-01TD | NA | DNA CLL | NA | Male | CLL | 5854945037 | R02C01 | EGAD00010000254 |
| 241 | 018-0046-01TD | NA | DNA CLL | NA | Male | CLL | 5854945039 | R02C01 | EGAD00010000254 |
| 242 | 019-0047-01TD | NA | DNA CLL | NA | Male | CLL | 5854945059 | R02C01 | EGAD00010000254 |
| 243 | 020-0049-01TD | NA | DNA CLL | NA | Male | CLL | 5822986021 | R03C01 | EGAD00010000254 |
| 244 | 022-0053-01TD | NA | DNA CLL | NA | Female | CLL | 5823031009 | R03C01 | EGAD00010000254 |
| 245 | 023-0056-01TD | NA | DNA CLL | NA | Male | CLL | 5823031011 | R03C01 | EGAD00010000254 |
| 246 | 024-0057-01TD | NA | DNA CLL | NA | Male | CLL | 5823031021 | R03C01 | EGAD00010000254 |
| 247 | 025-0059-01TD | NA | DNA CLL | NA | Female | CLL | 5854945025 | R03C01 | EGAD00010000254 |
| 248 | 026-0061-01TD | NA | DNA CLL | NA | Male | CLL | 5854945037 | R03C01 | EGAD00010000254 |
| 249 | 027-0063-01TD | NA | DNA CLL | NA | Male | CLL | 5854945039 | R03C01 | EGAD00010000254 |
| 250 | 029-0065-01TD | NA | DNA CLL | NA | Female | CLL | 5854945059 | R03C01 | EGAD00010000254 |
| 251 | 030-0066-01TD | NA | DNA CLL | NA | Male | CLL | 5822986021 | R04C01 | EGAD00010000254 |
| 252 | 032-0069-01TD | NA | DNA CLL | NA | Male | CLL | 5823031009 | R04C01 | EGAD00010000254 |
| 253 | 033-0070-01TD | NA | DNA CLL | NA | Male | CLL | 5823031011 | R04C01 | EGAD00010000254 |
| 254 | 037-02-1TD | NA | DNA CLL | NA | Female | CLL | 5823031021 | R04C01 | EGAD00010000254 |
| 255 | 039-0076-01TD | NA | DNA CLL | NA | Male | CLL | 5854945037 | R04C01 | EGAD00010000254 |
| 256 | 040-0088-01TD | NA | DNA CLL | NA | Female | CLL | 5854945039 | R04C01 | EGAD00010000254 |
| 257 | 041-0090-01TD | NA | DNA CLL | NA | Male | CLL | 5854945059 | R04C01 | EGAD00010000254 |
| 258 | 043-0094-01TD | NA | DNA CLL | NA | Male | CLL | 5822986021 | R05C01 | EGAD00010000254 |
| 259 | 044-0092-01TD | NA | DNA CLL | NA | Male | CLL | 5823031009 | R05C01 | EGAD00010000254 |
| 260 | 045-0082-04TD | NA | DNA CLL | NA | Female | CLL | 5823031011 | R05C01 | EGAD00010000254 |
| 261 | 046-0083-03TD | NA | DNA CLL | NA | Male | CLL | 5823031021 | R05C01 | EGAD00010000254 |
| 262 | 047-0084-06TD | NA | DNA CLL | NA | Male | CLL | 5854945025 | R05C01 | EGAD00010000254 |
| 263 | 048-0089-01TD | NA | DNA CLL | NA | Male | CLL | 5854945037 | R05C01 | EGAD00010000254 |
| 264 | 049-0086-01TD | NA | DNA CLL | NA | Female | CLL | 5854945039 | R05C01 | EGAD00010000254 |
| 265 | 051-0099-05TD | NA | DNA CLL | NA | Male | CLL | 5854945059 | R05C01 | EGAD00010000254 |
| 266 | 052-0103-01TD | NA | DNA CLL | NA | Male | CLL | 5822986021 | R06C01 | EGAD00010000254 |
| 267 | 053-0104-02TD | NA | DNA CLL | NA | Male | CLL | 5823031009 | R06C01 | EGAD00010000254 |
| 268 | 056-0117-01TD | NA | DNA CLL | NA | Female | CLL | 5823031011 | R06C01 | EGAD00010000254 |
| 269 | 063-0127-01TD | NA | DNA CLL | NA | Male | CLL | 5823031021 | R06C01 | EGAD00010000254 |
| 270 | 064-0128-01TD | NA | DNA CLL | NA | Male | CLL | 5854945025 | R06C01 | EGAD00010000254 |
| 271 | 082-02-1TD | NA | DNA CLL | NA | Female | CLL | 5854945037 | R06C01 | EGAD00010000254 |
| 272 | 083-01-2TD | NA | DNA CLL | NA | Female | CLL | 5854945039 | R06C01 | EGAD00010000254 |
| 273 | 090-02-1TD | NA | DNA CLL | NA | Female | CLL | 5854945059 | R06C01 | EGAD00010000254 |
| 274 | 100-02-2TD | NA | DNA CLL | NA | Male | CLL | 5822986021 | R01C02 | EGAD00010000254 |
| 275 | 109-0217-01TD | NA | DNA CLL | NA | Male | CLL | 5823031009 | R01C02 | EGAD00010000254 |
| 276 | 110-0218-04TD | NA | DNA CLL | NA | Female | CLL | 5823031011 | R01C02 | EGAD00010000254 |
| 277 | 117-01-1TD | NA | DNA CLL | NA | Female | CLL | 5823031021 | R01C02 | EGAD00010000254 |
| 278 | 124-01-1TD | NA | DNA CLL | NA | Male | CLL | 5854945025 | R01C02 | EGAD00010000254 |
| 279 | 128-03-2TD | NA | DNA CLL | NA | Male | CLL | 5854945037 | R01C02 | EGAD00010000254 |
| 280 | 131-02-5TD | NA | DNA CLL | NA | Male | CLL | 5854945039 | R01C02 | EGAD00010000254 |
| 281 | 136-02-3TD | NA | DNA CLL | NA | Male | CLL | 5854945059 | R01C02 | EGAD00010000254 |
| 282 | 138-03-2TD | NA | DNA CLL | NA | Female | CLL | 5822986021 | R02C02 | EGAD00010000254 |
| 283 | 144-01-1TD | NA | DNA CLL | NA | Male | CLL | 5823031009 | R02C02 | EGAD00010000254 |
| 284 | 146-01-5TD | NA | DNA CLL | NA | Male | CLL | 5823031021 | R02C02 | EGAD00010000254 |
| 285 | 147-02-3TD | NA | DNA CLL | NA | Male | CLL | 5854945025 | R02C02 | EGAD00010000254 |
| 286 | 152-01-4TD | NA | DNA CLL | NA | Male | CLL | 5854945037 | R02C02 | EGAD00010000254 |
| 287 | 155-01-1TD | NA | DNA CLL | NA | Female | CLL | 5854945039 | R02C02 | EGAD00010000254 |
| 288 | 156-01-1TD | NA | DNA CLL | NA | Male | CLL | 5854945059 | R02C02 | EGAD00010000254 |
| 289 | 157-01-1TD | NA | DNA CLL | NA | Male | CLL | 5822986021 | R03C02 | EGAD00010000254 |
| 290 | 159-01-1TD | NA | DNA CLL | NA | Male | CLL | 5823031009 | R03C02 | EGAD00010000254 |
| 291 | 166-01-4TD | NA | DNA CLL | NA | Male | CLL | 5823031011 | R03C02 | EGAD00010000254 |
| 292 | 168-02-2TD | NA | DNA CLL | NA | Male | CLL | 5823031021 | R03C02 | EGAD00010000254 |
| 293 | 170-01-3TD | NA | DNA CLL | NA | Male | CLL | 5854945025 | R03C02 | EGAD00010000254 |
| 294 | 171-01-2TD | NA | DNA CLL | NA | Female | CLL | 5854945037 | R03C02 | EGAD00010000254 |
| 295 | 172-01-1TD | NA | DNA CLL | NA | Female | CLL | 5854945039 | R03C02 | EGAD00010000254 |
| 296 | 173-01-3TD | NA | DNA CLL | NA | Male | CLL | 5854945059 | R03C02 | EGAD00010000254 |
| 297 | 174-01-3TD | NA | DNA CLL | NA | Female | CLL | 5822986021 | R04C02 | EGAD00010000254 |
| 298 | 175-01-3TD | NA | DNA CLL | NA | Male | CLL | 5823031009 | R04C02 | EGAD00010000254 |
| 299 | 180-02-1TD | NA | DNA CLL | NA | Female | CLL | 5823031011 | R04C02 | EGAD00010000254 |
| 300 | 181-01-3TD | NA | DNA CLL | NA | Male | CLL | 5823031021 | R04C02 | EGAD00010000254 |
| 301 | 182-01-4TD | NA | DNA CLL | NA | Male | CLL | 5854945025 | R04C02 | EGAD00010000254 |
| 302 | 184-01-4TD | NA | DNA CLL | NA | Male | CLL | 5854945037 | R04C02 | EGAD00010000254 |
| 303 | 185-01-6TD | NA | DNA CLL | NA | Male | CLL | 5854945039 | R04C02 | EGAD00010000254 |
| 304 | 186-01-6TD | NA | DNA CLL | NA | Male | CLL | 5854945059 | R04C02 | EGAD00010000254 |
| 305 | 188-01-2TD | NA | DNA CLL | NA | Male | CLL | 5822986021 | R05C02 | EGAD00010000254 |
| 306 | 189-01-1TD | NA | DNA CLL | NA | Female | CLL | 5823031009 | R05C02 | EGAD00010000254 |
| 307 | 191-01-3TD | NA | DNA CLL | NA | Male | CLL | 5823031011 | R05C02 | EGAD00010000254 |
| 308 | 192-01-4TD | NA | DNA CLL | NA | Female | CLL | 5823031021 | R05C02 | EGAD00010000254 |
| 309 | 193-01-1TD | NA | DNA CLL | NA | Female | CLL | 5854945025 | R05C02 | EGAD00010000254 |
| 310 | 194-01-2TD | NA | DNA CLL | NA | Female | CLL | 5854945037 | R05C02 | EGAD00010000254 |
| 311 | 195-01-5TD | NA | DNA CLL | NA | Male | CLL | 5854945039 | R05C02 | EGAD00010000254 |
| 312 | 197-01-3TD | NA | DNA CLL | NA | Female | CLL | 5854945059 | R05C02 | EGAD00010000254 |
| 313 | 264-01-7TD | NA | DNA CLL | NA | Male | CLL | 5822986021 | R06C02 | EGAD00010000254 |
| 314 | 267-01-6TD | NA | DNA CLL | NA | Female | CLL | 5823031009 | R06C02 | EGAD00010000254 |
| 315 | 270-01-2TD | NA | DNA CLL | NA | Male | CLL | 5823031011 | R06C02 | EGAD00010000254 |
| 316 | 273-01-5TD | NA | DNA CLL | NA | Male | CLL | 5823031021 | R06C02 | EGAD00010000254 |
| 317 | 274-01-3TD | NA | DNA CLL | NA | Male | CLL | 5854945025 | R06C02 | EGAD00010000254 |
| 318 | 275-01-2TD | NA | DNA CLL | NA | Female | CLL | 5854945037 | R06C02 | EGAD00010000254 |
| 319 | 276-01-4TD | NA | DNA CLL | NA | Male | CLL | 5854945039 | R06C02 | EGAD00010000254 |
| 320 | 277-01-3TD | NA | DNA CLL | NA | Female | CLL | 5854945059 | R06C02 | EGAD00010000254 |
| 321 | 278-01-4TD | NA | DNA CLL | NA | Male | CLL | 5859594001 | R01C01 | EGAD00010000254 |
| 322 | 280-01-4TD | NA | DNA CLL | NA | Female | CLL | 5859594003 | R01C01 | EGAD00010000254 |
| 323 | 282-01-12TD | NA | DNA CLL | NA | Female | CLL | 5859594004 | R01C01 | EGAD00010000254 |
| 324 | 290-1950-01TD | NA | DNA CLL | NA | Female | CLL | 5859594010 | R01C01 | EGAD00010000254 |
| 325 | 294-01-3TD | NA | DNA CLL | NA | Male | CLL | 5859594011 | R01C01 | EGAD00010000254 |
| 326 | 297-01-1TD | NA | DNA CLL | NA | Male | CLL | 5859594013 | R01C01 | EGAD00010000254 |
| 327 | 298-01-5TD | NA | DNA CLL | NA | Male | CLL | 5859594014 | R01C01 | EGAD00010000254 |
| 328 | 305-01-5TD | NA | DNA CLL | NA | Male | CLL | 5859594030 | R01C01 | EGAD00010000254 |
| 329 | 306-01-4TD | NA | DNA CLL | NA | Male | CLL | 5859594001 | R02C01 | EGAD00010000254 |
| 330 | 308-01-4TD | NA | DNA CLL | NA | Male | CLL | 5859594003 | R02C01 | EGAD00010000254 |
| 331 | 312-01-5TD | NA | DNA CLL | NA | Female | CLL | 5859594004 | R02C01 | EGAD00010000254 |
| 332 | 313-01-7TD | NA | DNA CLL | NA | Female | CLL | 5859594010 | R02C01 | EGAD00010000254 |
| 333 | 315-01-7TD | NA | DNA CLL | NA | Female | CLL | 5859594011 | R02C01 | EGAD00010000254 |
| 334 | 316-01-8TD | NA | DNA CLL | NA | Male | CLL | 5859594013 | R02C01 | EGAD00010000254 |
| 335 | 318-01-2TD | NA | DNA CLL | NA | Female | CLL | 5859594014 | R02C01 | EGAD00010000254 |
| 336 | 319-01-1TD | NA | DNA CLL | NA | Male | CLL | 5859594030 | R02C01 | EGAD00010000254 |
| 337 | 322-01-1TD | NA | DNA CLL | NA | Male | CLL | 5859594001 | R03C01 | EGAD00010000254 |
| 338 | 323-01-1TD | NA | DNA CLL | NA | Female | CLL | 5859594003 | R03C01 | EGAD00010000254 |
| 339 | 324-01-1TD | NA | DNA CLL | NA | Female | CLL | 5859594004 | R03C01 | EGAD00010000254 |
| 340 | 325-01-1TD | NA | DNA CLL | NA | Female | CLL | 5859594010 | R03C01 | EGAD00010000254 |
| 341 | 326-01-1TD | NA | DNA CLL | NA | Female | CLL | 5859594011 | R03C01 | EGAD00010000254 |
| 342 | 330-01-1TD | NA | DNA CLL | NA | Male | CLL | 5859594014 | R03C01 | EGAD00010000254 |
| 343 | 331-01-3TD | NA | DNA CLL | NA | Female | CLL | 5859594030 | R03C01 | EGAD00010000254 |
| 344 | 338-01-5TD | NA | DNA CLL | NA | Female | CLL | 5859594001 | R04C01 | EGAD00010000254 |
| 345 | 341-01-2TD | NA | DNA CLL | NA | Male | CLL | 5859594003 | R04C01 | EGAD00010000254 |
| 346 | 342-01-2TD | NA | DNA CLL | NA | Male | CLL | 5859594004 | R04C01 | EGAD00010000254 |
| 347 | 344-01-7TD | NA | DNA CLL | NA | Female | CLL | 5859594010 | R04C01 | EGAD00010000254 |
| 348 | 345-01-3TD | NA | DNA CLL | NA | Female | CLL | 5859594011 | R04C01 | EGAD00010000254 |
| 349 | 350-01-1TD | NA | DNA CLL | NA | Male | CLL | 5859594013 | R04C01 | EGAD00010000254 |
| 350 | 351-01-4TD | NA | DNA CLL | NA | Female | CLL | 5859594014 | R04C01 | EGAD00010000254 |
| 351 | 358-01-2TD | NA | DNA CLL | NA | Male | CLL | 5859594030 | R04C01 | EGAD00010000254 |
| 352 | 371-01-6TD | NA | DNA CLL | NA | Female | CLL | 5859594003 | R05C01 | EGAD00010000254 |
| 353 | 373-01-5TD | NA | DNA CLL | NA | Female | CLL | 5859594004 | R05C01 | EGAD00010000254 |
| 354 | 375-1099-15TD | NA | DNA CLL | NA | Male | CLL | 5859594010 | R05C01 | EGAD00010000254 |
| 355 | 475-02-10TD | NA | DNA CLL | NA | Female | CLL | 5859594011 | R05C01 | EGAD00010000254 |
| 356 | 535-02-4TD | NA | DNA CLL | NA | Male | CLL | 5859594013 | R05C01 | EGAD00010000254 |
| 357 | 597-01-2TD | NA | DNA CLL | NA | Male | CLL | 5859594014 | R05C01 | EGAD00010000254 |
| 358 | 618-1503-04TD | NA | DNA CLL | NA | Male | CLL | 5859594030 | R05C01 | EGAD00010000254 |
| 359 | 680-1992-01TD | NA | DNA CLL | NA | Male | CLL | 5859594001 | R06C01 | EGAD00010000254 |
| 360 | 723-03-2TD | NA | DNA CLL | NA | Male | CLL | 5859594003 | R06C01 | EGAD00010000254 |
| 361 | 761-01-1TD | NA | DNA CLL | NA | Male | CLL | 5859594004 | R06C01 | EGAD00010000254 |
| 362 | 785-1836-01TD | NA | DNA CLL | NA | Male | CLL | 5859594010 | R06C01 | EGAD00010000254 |
| 363 | 815-01-2TD | NA | DNA CLL | NA | Male | CLL | 5859594011 | R06C01 | EGAD00010000254 |
| 364 | 866-2008-01TD | NA | DNA CLL | NA | Male | CLL | 5859594013 | R06C01 | EGAD00010000254 |
| 365 | 876-2035-16TD | NA | DNA CLL | NA | Female | CLL | 5859594014 | R06C01 | EGAD00010000254 |
| 366 | 901.01.1D | NA | DNA control | NA | Male | B cell | 5859594014 | R03C02 | EGAD00010000254 |
| 367 | 902.01.1D | NA | DNA control | NA | Male | B cell | 5859594030 | R03C02 | EGAD00010000254 |
| 368 | 903.01.1D | NA | DNA control | NA | Female | B cell | 5859594001 | R04C02 | EGAD00010000254 |
| 369 | 904.01.1D | NA | DNA control | NA | Female | B cell | 5859594003 | R04C02 | EGAD00010000254 |
| 370 | 905.01.1D | NA | DNA control | NA | Male | B cell | 5859594004 | R04C02 | EGAD00010000254 |
| 371 | 908.01.1D | NA | DNA control | NA | Female | B cell | 5859594010 | R04C02 | EGAD00010000254 |
| 372 | 909.01.1D | NA | DNA control | NA | Female | B cell | 5859594011 | R04C02 | EGAD00010000254 |
| 373 | 913.01.1D | NA | DNA control | NA | Female | B cell | 5859594014 | R04C02 | EGAD00010000254 |
| 374 | 915.01.1D | NA | DNA control | NA | Female | B cell | 5859594030 | R04C02 | EGAD00010000254 |
| 375 | 918.01.1D | NA | DNA control | NA | Male | B cell | 5859594001 | R05C02 | EGAD00010000254 |
| 376 | 919.01.1D | NA | DNA control | NA | Male | B cell | 5859594003 | R05C02 | EGAD00010000254 |
| 377 | 920.01.1D | NA | DNA control | NA | Male | B cell | 5859594004 | R05C02 | EGAD00010000254 |
| 378 | 924.01.1D | NA | DNA control | NA | Male | B cell | 5859594010 | R05C02 | EGAD00010000254 |
| 379 | 925.01.1D | NA | DNA control | NA | Male | B cell | 5859594011 | R05C02 | EGAD00010000254 |
| 380 | 932.01.4D | NA | DNA control | NA | Male | NaiveB | 5900833028 | R01C02 | EGAD00010000254 |
| 381 | 932.01.5D | NA | DNA control | NA | Male | Non-Class-switched-MemoryB | 5900833028 | R02C02 | EGAD00010000254 |
| 382 | 932.01.6D | NA | DNA control | NA | Male | Class-switched-MemoryB | 5900833028 | R03C02 | EGAD00010000254 |
| 383 | 938.01.1D | NA | DNA control | NA | Female | NaiveB | 5900833028 | R01C01 | EGAD00010000254 |
| 384 | 938.01.2D | NA | DNA control | NA | Female | Non-Class-switched-MemoryB | 5900833028 | R02C01 | EGAD00010000254 |
| 385 | 938.01.3D | NA | DNA control | NA | Female | Class-switched-MemoryB | 5900833028 | R03C01 | EGAD00010000254 |
| 386 | 939.01.1D | NA | DNA control | NA | Female | NaiveB | 5900833028 | R04C01 | EGAD00010000254 |
| 387 | 939.01.2D | NA | DNA control | NA | Female | Non-Class-switched-MemoryB | 5900833028 | R05C01 | EGAD00010000254 |
| 388 | 939.01.3D | NA | DNA control | NA | Female | Class-switched-MemoryB | 5900833028 | R06C01 | EGAD00010000254 |
| 389 | 940.01.1D | NA | DNA control | NA | Female | CD5+ NaiveB | 5900833028 | R04C02 | EGAD00010000254 |
| 390 | 941.01.1D | NA | DNA control | NA | Female | CD5+ NaiveB | 5900833028 | R05C02 | EGAD00010000254 |
| 391 | 942.01.1D | NA | DNA control | NA | Male | CD5+ NaiveB | 5900833028 | R06C02 | EGAD00010000254 |

Supplementary Table S2C. RNASeq data of CLL cases (EGAD00001000258 and GSE66117), and healthy control (GSE62246and GSE70830)

|  | Sample | Didease | Platform | Study |
| --- | --- | --- | --- | --- |
| 1 | 626WAAAXX_1 | CLL | Illumina HiSeq 2000 | EGAD00001000258 |
| 2 | 626WAAAXX_2 | CLL | Illumina HiSeq 2000 | EGAD00001000258 |
| 3 | 626WAAAXX_3 | CLL | Illumina HiSeq 2000 | EGAD00001000258 |
| 4 | 626WAAAXX_4 | CLL | Illumina HiSeq 2000 | EGAD00001000258 |
| 5 | 626WGAAXX_1 | CLL | Illumina HiSeq 2000 | EGAD00001000258 |
| 6 | 626WGAAXX_2 | CLL | Illumina HiSeq 2000 | EGAD00001000258 |
| 7 | 626WGAAXX_3 | CLL | Illumina HiSeq 2000 | EGAD00001000258 |
| 8 | 626WGAAXX_4 | CLL | Illumina HiSeq 2000 | EGAD00001000258 |
| 9 | 626WGAAXX_7 | CLL | Illumina HiSeq 2000 | EGAD00001000258 |
| 10 | 626WGAAXX_8 | CLL | Illumina HiSeq 2000 | EGAD00001000258 |
| 11 | 627LPAAXX_1 | CLL | Illumina HiSeq 2000 | EGAD00001000258 |
| 12 | 627LPAAXX_2 | CLL | Illumina HiSeq 2000 | EGAD00001000258 |
| 13 | 627LPAAXX_3 | CLL | Illumina HiSeq 2000 | EGAD00001000258 |
| 14 | 627LPAAXX_4 | CLL | Illumina HiSeq 2000 | EGAD00001000258 |
| 15 | 627LPAAXX_7 | CLL | Illumina HiSeq 2000 | EGAD00001000258 |
| 16 | 627LPAAXX_8 | CLL | Illumina HiSeq 2000 | EGAD00001000258 |
| 17 | 627M8AAXX_1 | CLL | Illumina HiSeq 2000 | EGAD00001000258 |
| 18 | 627M8AAXX_2 | CLL | Illumina HiSeq 2000 | EGAD00001000258 |
| 19 | 627M8AAXX_3 | CLL | Illumina HiSeq 2000 | EGAD00001000258 |
| 20 | 62JP4AAXX_1 | CLL | Illumina HiSeq 2000 | EGAD00001000258 |
| 21 | 62JP4AAXX_2 | CLL | Illumina HiSeq 2000 | EGAD00001000258 |
| 22 | 62JP4AAXX_8 | CLL | Illumina HiSeq 2000 | EGAD00001000258 |
| 23 | B02VRACXX_5_10 | CLL | Illumina HiSeq 2000 | EGAD00001000258 |
| 24 | B02VRACXX_5_11 | CLL | Illumina HiSeq 2000 | EGAD00001000258 |
| 25 | B02VRACXX_5_12 | CLL | Illumina HiSeq 2000 | EGAD00001000258 |
| 26 | B02VRACXX_5_9 | CLL | Illumina HiSeq 2000 | EGAD00001000258 |
| 27 | B02VRACXX_6_10 | CLL | Illumina HiSeq 2000 | EGAD00001000258 |
| 28 | B02VRACXX_6_11 | CLL | Illumina HiSeq 2000 | EGAD00001000258 |
| 29 | B02VRACXX_6_12 | CLL | Illumina HiSeq 2000 | EGAD00001000258 |
| 30 | B02VRACXX_6_9 | CLL | Illumina HiSeq 2000 | EGAD00001000258 |
| 31 | B02VRACXX_7_1 | CLL | Illumina HiSeq 2000 | EGAD00001000258 |
| 32 | B02VRACXX_7_2 | CLL | Illumina HiSeq 2000 | EGAD00001000258 |
| 33 | B02VRACXX_7_3 | CLL | Illumina HiSeq 2000 | EGAD00001000258 |
| 34 | B02VRACXX_7_4 | CLL | Illumina HiSeq 2000 | EGAD00001000258 |
| 35 | B02VRACXX_8_5 | CLL | Illumina HiSeq 2000 | EGAD00001000258 |
| 36 | B02VRACXX_8_6 | CLL | Illumina HiSeq 2000 | EGAD00001000258 |
| 37 | B02VRACXX_8_7 | CLL | Illumina HiSeq 2000 | EGAD00001000258 |
| 38 | B02VRACXX_8_8 | CLL | Illumina HiSeq 2000 | EGAD00001000258 |
| 39 | C01BRACXX_4_2 | CLL | Illumina HiSeq 2000 | EGAD00001000258 |
| 40 | C01BRACXX_4_3 | CLL | Illumina HiSeq 2000 | EGAD00001000258 |
| 41 | C01BRACXX_5_4 | CLL | Illumina HiSeq 2000 | EGAD00001000258 |
| 42 | C01BRACXX_5_5 | CLL | Illumina HiSeq 2000 | EGAD00001000258 |
| 43 | C01BRACXX_5_6 | CLL | Illumina HiSeq 2000 | EGAD00001000258 |
| 44 | C01BRACXX_6_7 | CLL | Illumina HiSeq 2000 | EGAD00001000258 |
| 45 | C01BRACXX_6_8 | CLL | Illumina HiSeq 2000 | EGAD00001000258 |
| 46 | C01BRACXX_6_9 | CLL | Illumina HiSeq 2000 | EGAD00001000258 |
| 47 | C01BRACXX_7_10 | CLL | Illumina HiSeq 2000 | EGAD00001000258 |
| 48 | C01BRACXX_7_11 | CLL | Illumina HiSeq 2000 | EGAD00001000258 |
| 49 | C01BRACXX_7_12 | CLL | Illumina HiSeq 2000 | EGAD00001000258 |
| 50 | C01BRACXX_8_1 | CLL | Illumina HiSeq 2000 | EGAD00001000258 |
| 51 | C01BRACXX_8_2 | CLL | Illumina HiSeq 2000 | EGAD00001000258 |
| 52 | C01BRACXX_8_3 | CLL | Illumina HiSeq 2000 | EGAD00001000258 |
| 53 | C01CLACXX_7_1 | CLL | Illumina HiSeq 2000 | EGAD00001000258 |
| 54 | C01CLACXX_7_10 | CLL | Illumina HiSeq 2000 | EGAD00001000258 |
| 55 | C01CLACXX_7_11 | CLL | Illumina HiSeq 2000 | EGAD00001000258 |
| 56 | C01CLACXX_7_12 | CLL | Illumina HiSeq 2000 | EGAD00001000258 |
| 57 | C01CLACXX_8_1 | CLL | Illumina HiSeq 2000 | EGAD00001000258 |
| 58 | C01CLACXX_8_2 | CLL | Illumina HiSeq 2000 | EGAD00001000258 |
| 59 | C01CLACXX_8_3 | CLL | Illumina HiSeq 2000 | EGAD00001000258 |
| 60 | C01CLACXX_8_4 | CLL | Illumina HiSeq 2000 | EGAD00001000258 |
| 61 | C0EWEACXX_3_19 | CLL | Illumina HiSeq 2000 | EGAD00001000258 |
| 62 | CLL250909_7 | CLL | Illumina HiSeq 2000 | EGAD00001000258 |
| 63 | CLL280809_7 | CLL | Illumina HiSeq 2000 | EGAD00001000258 |
| 64 | D030UACXX_7_1 | CLL | Illumina HiSeq 2000 | EGAD00001000258 |
| 65 | D030UACXX_7_2 | CLL | Illumina HiSeq 2000 | EGAD00001000258 |
| 66 | D030UACXX_7_3 | CLL | Illumina HiSeq 2000 | EGAD00001000258 |
| 67 | D030UACXX_7_4 | CLL | Illumina HiSeq 2000 | EGAD00001000258 |
| 68 | D030UACXX_8_5 | CLL | Illumina HiSeq 2000 | EGAD00001000258 |
| 69 | D030UACXX_8_6 | CLL | Illumina HiSeq 2000 | EGAD00001000258 |
| 70 | D030UACXX_8_7 | CLL | Illumina HiSeq 2000 | EGAD00001000258 |
| 71 | D030UACXX_8_8 | CLL | Illumina HiSeq 2000 | EGAD00001000258 |
| 72 | D0933ACXX_1_1 | CLL | Illumina HiSeq 2000 | EGAD00001000258 |
| 73 | D0933ACXX_1_2 | CLL | Illumina HiSeq 2000 | EGAD00001000258 |
| 74 | D0933ACXX_1_3 | CLL | Illumina HiSeq 2000 | EGAD00001000258 |
| 75 | D0933ACXX_1_4 | CLL | Illumina HiSeq 2000 | EGAD00001000258 |
| 76 | D0933ACXX_2_2 | CLL | Illumina HiSeq 2000 | EGAD00001000258 |
| 77 | D0933ACXX_2_3 | CLL | Illumina HiSeq 2000 | EGAD00001000258 |
| 78 | D0933ACXX_2_4 | CLL | Illumina HiSeq 2000 | EGAD00001000258 |
| 79 | D0933ACXX_3_1 | CLL | Illumina HiSeq 2000 | EGAD00001000258 |
| 80 | D0933ACXX_3_5 | CLL | Illumina HiSeq 2000 | EGAD00001000258 |
| 81 | D0933ACXX_3_6 | CLL | Illumina HiSeq 2000 | EGAD00001000258 |
| 82 | D0933ACXX_3_7 | CLL | Illumina HiSeq 2000 | EGAD00001000258 |
| 83 | D0933ACXX_5_10 | CLL | Illumina HiSeq 2000 | EGAD00001000258 |
| 84 | D0933ACXX_5_2 | CLL | Illumina HiSeq 2000 | EGAD00001000258 |
| 85 | D0933ACXX_5_8 | CLL | Illumina HiSeq 2000 | EGAD00001000258 |
| 86 | D0933ACXX_5_9 | CLL | Illumina HiSeq 2000 | EGAD00001000258 |
| 87 | D0933ACXX_6_11 | CLL | Illumina HiSeq 2000 | EGAD00001000258 |
| 88 | D0933ACXX_6_5 | CLL | Illumina HiSeq 2000 | EGAD00001000258 |
| 89 | D0933ACXX_6_6 | CLL | Illumina HiSeq 2000 | EGAD00001000258 |
| 90 | D0933ACXX_6_7 | CLL | Illumina HiSeq 2000 | EGAD00001000258 |
| 91 | D0933ACXX_7_1 | CLL | Illumina HiSeq 2000 | EGAD00001000258 |
| 92 | D0933ACXX_7_12 | CLL | Illumina HiSeq 2000 | EGAD00001000258 |
| 93 | D0933ACXX_7_2 | CLL | Illumina HiSeq 2000 | EGAD00001000258 |
| 94 | D0933ACXX_7_8 | CLL | Illumina HiSeq 2000 | EGAD00001000258 |
| 95 | D0933ACXX_8_3 | CLL | Illumina HiSeq 2000 | EGAD00001000258 |
| 96 | D0933ACXX_8_4 | CLL | Illumina HiSeq 2000 | EGAD00001000258 |
| 97 | D0933ACXX_8_5 | CLL | Illumina HiSeq 2000 | EGAD00001000258 |
| 98 | D0933ACXX_8_9 | CLL | Illumina HiSeq 2000 | EGAD00001000258 |
| 99 | SRR1812702 | CLL | Illumina HiSeq 2000 | GSE66167 |
| 100 | SRR1812703 | CLL | Illumina HiSeq 2000 | GSE66167 |
| 101 | SRR1812704 | CLL | Illumina HiSeq 2000 | GSE66167 |
| 102 | SRR1812705 | CLL | Illumina HiSeq 2000 | GSE66167 |
| 103 | SRR1812706 | CLL | Illumina HiSeq 2000 | GSE66167 |
| 104 | SRR1812707 | CLL | Illumina HiSeq 2000 | GSE66167 |
| 105 | SRR1812708 | CLL | Illumina HiSeq 2000 | GSE66167 |
| 106 | SRR1812709 | CLL | Illumina HiSeq 2000 | GSE66167 |
| 107 | SRR1812710 | CLL | Illumina HiSeq 2000 | GSE66167 |
| 108 | SRR1812711 | CLL | Illumina HiSeq 2000 | GSE66167 |
| 109 | SRR1812712 | CLL | Illumina HiSeq 2000 | GSE66167 |
| 110 | SRR1812713 | CLL | Illumina HiSeq 2000 | GSE66167 |
| 111 | SRR1812714 | CLL | Illumina HiSeq 2000 | GSE66167 |
| 112 | SRR1812715 | CLL | Illumina HiSeq 2000 | GSE66167 |
| 113 | SRR1812716 | CLL | Illumina HiSeq 2000 | GSE66167 |
| 114 | SRR1812717 | CLL | Illumina HiSeq 2000 | GSE66167 |
| 115 | SRR1812718 | CLL | Illumina HiSeq 2000 | GSE66167 |
| 116 | SRR1812719 | CLL | Illumina HiSeq 2000 | GSE66167 |
| 117 | SRR1812720 | CLL | Illumina HiSeq 2000 | GSE66167 |
| 118 | SRR1812721 | CLL | Illumina HiSeq 2000 | GSE66167 |
| 119 | SRR1812722 | CLL | Illumina HiSeq 2000 | GSE66167 |
| 120 | SRR1812723 | CLL | Illumina HiSeq 2000 | GSE66167 |
| 121 | SRR1812724 | CLL | Illumina HiSeq 2000 | GSE66167 |
| 122 | SRR1812725 | CLL | Illumina HiSeq 2000 | GSE66167 |
| 123 | SRR1812726 | CLL | Illumina HiSeq 2000 | GSE66167 |
| 124 | SRR1812727 | CLL | Illumina HiSeq 2000 | GSE66167 |
| 125 | SRR1812728 | CLL | Illumina HiSeq 2000 | GSE66167 |
| 126 | SRR1812729 | CLL | Illumina HiSeq 2000 | GSE66167 |
| 127 | SRR1812730 | CLL | Illumina HiSeq 2000 | GSE66167 |
| 128 | SRR1812731 | CLL | Illumina HiSeq 2000 | GSE66167 |
| 129 | SRR1812732 | CLL | Illumina HiSeq 2000 | GSE66167 |
| 130 | SRR1812733 | CLL | Illumina HiSeq 2000 | GSE66167 |
| 131 | SRR1812734 | CLL | Illumina HiSeq 2000 | GSE66167 |
| 132 | SRR1812735 | CLL | Illumina HiSeq 2000 | GSE66167 |
| 133 | SRR1812736 | CLL | Illumina HiSeq 2000 | GSE66167 |
| 134 | SRR1812737 | CLL | Illumina HiSeq 2000 | GSE66167 |
| 135 | SRR1812738 | CLL | Illumina HiSeq 2000 | GSE66167 |
| 136 | SRR1812739 | CLL | Illumina HiSeq 2000 | GSE66167 |
| 137 | SRR1812740 | CLL | Illumina HiSeq 2000 | GSE66167 |
| 138 | SRR1812741 | CLL | Illumina HiSeq 2000 | GSE66167 |
| 139 | SRR1812742 | CLL | Illumina HiSeq 2000 | GSE66167 |
| 140 | SRR1812743 | CLL | Illumina HiSeq 2000 | GSE66167 |
| 141 | SRR1812744 | CLL | Illumina HiSeq 2000 | GSE66167 |
| 142 | SRR1812745 | CLL | Illumina HiSeq 2000 | GSE66167 |
| 143 | SRR1812746 | CLL | Illumina HiSeq 2000 | GSE66167 |
| 144 | SRR1812747 | CLL | Illumina HiSeq 2000 | GSE66167 |
| 145 | SRR1812748 | CLL | Illumina HiSeq 2000 | GSE66167 |
| 146 | SRR1609991 | Normal | Illumina HiSeq 2000 | GSE62246 |
| 147 | SRR1609992 | Normal | Illumina HiSeq 2000 | GSE62246 |
| 148 | SRR2097512 | Normal | Illumina HiSeq 2000 | GSE70830 |
| 149 | SRR2097513 | Normal | Illumina HiSeq 2000 | GSE70830 |
| 150 | SRR2097514 | Normal | Illumina HiSeq 2000 | GSE70830 |
| 151 | SRR2097515 | Normal | Illumina HiSeq 2000 | GSE70830 |
| 152 | SRR2097516 | Normal | Illumina HiSeq 2000 | GSE70830 |
